# Human *VCP* mutant ALS/FTD microglia display immune and lysosomal phenotypes independently of GPNMB

**DOI:** 10.1186/s13024-024-00773-1

**Published:** 2024-11-26

**Authors:** Benjamin E. Clarke, Oliver J. Ziff, Giulia Tyzack, Marija Petrić Howe, Yiran Wang, Pierre Klein, Claudia A. Smith, Cameron A. Hall, Adel Helmy, Michael Howell, Gavin Kelly, Rickie Patani

**Affiliations:** 1 Department of Neuromuscular Diseases, Queen Square Institute of Neurology, University College London, London, WC1N 3BG UK; 2 The Francis Crick Institute, 1 Midland Road, London, NW1 1AT UK; 3 National Hospital for Neurology and Neurosurgery, University College London NHS Foundation Trust, London, WC1N 3BG UK; 4 Division of Neurosurgery and Wolfson Brain Imaging Centre, Department of Clinical Neurosciences, University of Cambridge, Cambridge, UK

## Abstract

**Background:**

Microglia play crucial roles in maintaining neuronal homeostasis but have been implicated in contributing to amyotrophic lateral sclerosis (ALS) and frontotemporal dementia (FTD). However, the role of microglia in ALS/FTD remains incompletely understood.

**Methods:**

Here, we generated highly enriched cultures of *VCP* mutant microglia derived from human induced pluripotent stem cells (hiPSCs) to investigate their cell autonomous and non-cell autonomous roles in ALS pathogenesis. We used RNA-sequencing, proteomics and functional assays to study hiPSC derived *VCP* mutant microglia and their effects on hiPSC derived motor neurons and astrocytes.

**Results:**

Transcriptomic, proteomic and functional analyses revealed immune and lysosomal dysfunction in *VCP* mutant microglia. Stimulating healthy microglia with the inflammatory inducer lipopolysaccharide (LPS) showed partial overlap with *VCP* mutant microglia in their reactive transformation. LPS-stimulated *VCP* mutant microglia displayed differential activation of inflammatory pathways compared with LPS-stimulated healthy microglia. Conserved gene expression changes were identified between *VCP* mutant microglia, *SOD1* mutant mice microglia, and postmortem ALS spinal cord microglial signatures, including increased expression of the transmembrane glycoprotein *GPNMB*. While knockdown of *GPNMB* affected inflammatory and phagocytosis processes in microglia, this was not sufficient to ameliorate cell autonomous phenotypes in *VCP* mutant microglia. Secreted factors from *VCP* mutant microglia were sufficient to activate the JAK-STAT pathway in hiPSC derived motor neurons and astrocytes.

**Conclusions:**

*VCP* mutant microglia undergo cell autonomous reactive transformation involving immune and lysosomal dysfunction that partially recapitulate key phenotypes of microglia from other ALS models and post mortem tissue. These phenotypes occur independently of *GPNMB*. Additionally, *VCP* mutant microglia elicit non cell autonomous responses in motor neurons and astrocytes involving the JAK-STAT pathway.

**Supplementary Information:**

The online version contains supplementary material available at 10.1186/s13024-024-00773-1.

## Introduction

Amyotrophic lateral sclerosis (ALS) is a rapidly progressive and fatal neurodegenerative disease characterised by the loss of upper and lower motor neurons. Mutations in over 30 genes are causative of ALS, including *C9orf72*, *SOD1*, *FUS*, *TARDBP*, and *VCP* (valosin-containing protein*,* p97) [1, 2]. ALS exists on a clinical, genetic and pathological spectrum with frontotemporal dementia (FTD). 1–2% of familial ALS cases are caused by mutations in *VCP* [1]. VCP is an AAA + ATPase that interacts with several co-factors to perform an important role in protein homeostasis, which can, in turn, affect many cellular processes including lysosome function and clearance [3, 4], stress responses [5–7] and antiviral responses [8].

Microglia are the primary immune cells of the brain, with diverse roles in phagocytosis, neuronal excitability, synaptic maintenance, myelin homeostasis and vascular regulation [9]. In response to injury, infection, or disease, microglia undergo graded and context-dependent changes in their gene expression, morphology, and function [10, 11]. For example, microglia exposed to the gram-negative bacterial endotoxin, lipopolysaccharide (LPS), change their morphology and release cytokines [10, 12, 13]. While microglia can carry out acute responses to infection, they can also undergo chronic maladaptive changes in disease. Mounting evidence suggests that they drive neuroinflammation across a spectrum of neurodegenerative diseases and contribute to motor neuron death in ALS [14–17]. Altered microglial gene expression and morphology has been observed in postmortem spinal cord tissue of ALS patients and in *SOD1* mutant mouse models [18–24]. Furthermore, microglia play a modulatory role in disease progression in *SOD1* mutant mouse models once the disease has been initiated [17, 25–27]. While these studies point to a pathogenic role of microglia in the later stages of ALS, the precise contribution of microglia in the early stages of ALS pathogenesis remains less well understood.

Human induced pluripotent stem cells (hiPSCs) provide a valuable model system to study early disease phenotypes. hiPSC-derived microglia, which have not interacted with neuronal or other glial cell types, provide an opportunity to investigate their cell autonomous phenotypes. Furthermore, the study of human microglia is important due to species-specific differences, such as the expression of immune related genes (including TLR, Fcγ and SIGLEC receptors) [28, 29]. Recent studies have identified disease-specific phenotypes in hiPSC-derived ALS microglia, including endosomal-lysosomal, autophagy and immune response dysfunction in *C9orf72* mutant microglia [30–32] and chemoreceptor dysregulation in *FUS* mutant microglia [33].

While we have previously identified phenotypes in hiPSC-derived motor neurons and astrocytes from ALS patients with *VCP* mutations [34–37], the impact of *VCP* mutations on microglia remains unresolved. Here, we used an established protocol to generate highly pure cultures of hiPSC-derived microglia [38]. Through unbiased transcriptomics, proteomics and functional assays, we uncover cell autonomous changes in *VCP* mutant microglia and identify common signatures with post-mortem ALS, *SOD1* mutant mouse and LPS-stimulated microglia. These cell autonomous changes in *VCP* mutant microglia are sufficient to non-cell autonomously activate JAK-STAT in hiPSC-derived motor neurons and astrocytes. We found that transmembrane melanosome-associated glycoprotein (GPNMB) was upregulated across ALS models and is reported to be involved in inflammatory processes [39]. However, our study revealed that *VCP* mutant microglial phenotypes occur independently of *GPNMB*.

## Results

### hiPSC-derived microglia recapitulate gene expression of authentic human microglia

**Fig. 1:**
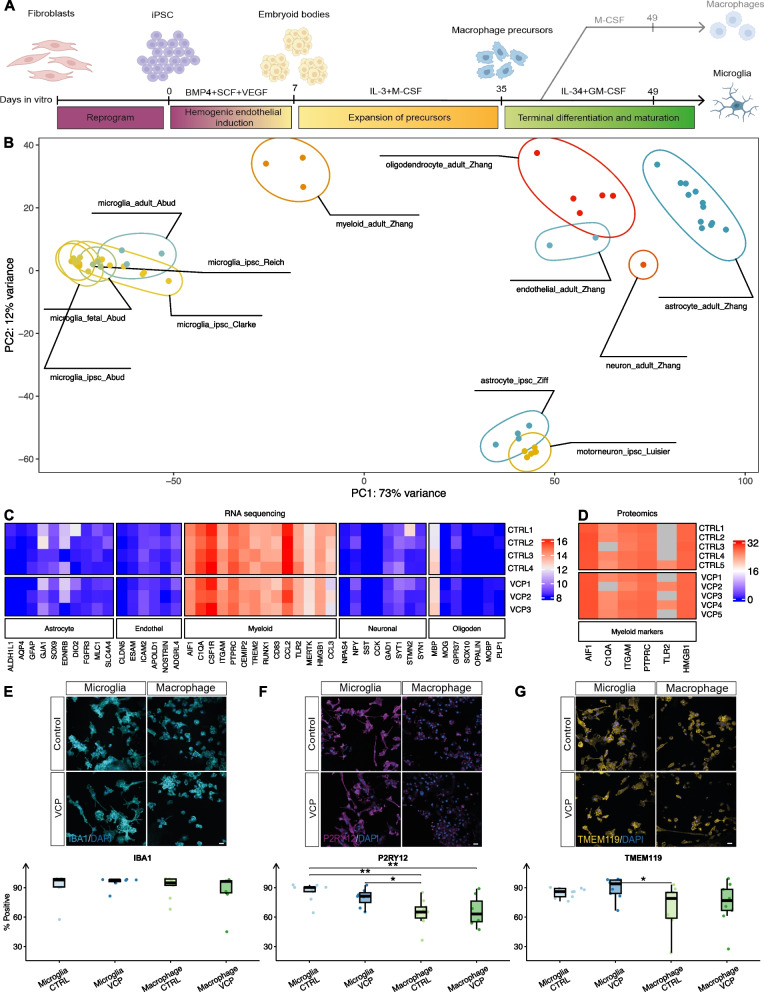
Characterisation of hiPSC-derived microglia cultures. **A** Schematic showing directed differentiation of hiPSCs to microglia and macrophages (termed hiPSC-derived macrophages and microglia hereafter). **B** Principal component analysis (PCA) of variance stabilised counts, plotted by their coordinates along the first two principal components of hiPSC microglia (current study denoted Clarke) as well as other publicly available hiPSC microglia [41, 42], other hiPSC derived CNS cell types [36, 43], primary microglia from fetal and adult human brain samples [41], and other primary CNS cell types from human brain samples [44]. **C** Heatmap showing RNA sequencing normalised gene expression counts of distinct CNS cell types across our healthy control and *VCP* mutant hiPSC-derived microglia. **D** Heatmap showing microglial protein marker intensities detected by mass spectrometry across our healthy control and *VCP* mutant hiPSC-derived microglia. Immunofluorescence images and quantification of (**E**) IBA1, **F** P2RY12 and (**G**) TMEM119. Scale bar: 20 μm. Wilcoxon-test was used to determine significance (** *P* < 0.01, * *P* < 0.05)

We generated hiPSC-derived microglia using a previously defined ontogeny-recapitulating protocol, which validated microglial identity through differentiation of yolk-sac derived *Myb*-independent progenitors[38, 40] (Fig. 1A). We utilised six healthy control lines and six lines carrying ALS-causing *VCP* mutations. To improve the detection of mutation-dependent changes, *VCP* mutant lines included two from ALS patients with native *VCP* mutations (VCP^R191Q^ and VCP^R155C^), two lines with the *VCP*^*R191Q*^ mutation inserted, and an isogenic line with the *VCP*^*R155C*^ mutation corrected (Fig. S1A, Table S1). The identity of our hiPSC microglial cultures was confirmed through poly(A) RNA sequencing (Table S2), mass spectrometry, and immunofluorescence for specific markers of cellular identity.

Principal Component Analysis (PCA) and unsupervised hierarchical clustering of RNA-seq gene expression demonstrated that our healthy control and *VCP* mutant hiPSC-derived microglia clustered together with other hiPSC-derived microglia datasets, as well as microglia from fetal and adult brain samples. Additionally, our hiPSC-derived microglia formed distinct clusters separate from other cell types including neurons, astrocytes, oligodendrocytes and endothelial cells[36, 41–44] (Fig. 1B; S1B). Our hiPSC-derived microglia were enriched in gene and protein expression of myeloid markers while displaying minimal expression of markers associated with other CNS cell types (Fig. 1C-D). We further assessed microglial identity by examining the comprehensive core human microglial transcriptional signature consisting of 249 high-confidence markers[45], which confirmed increased expression of microglial markers across our microglia samples compared to other CNS cell types isolated from human brain samples[44] (Fig. S1C).

We used quantitative immunofluorescence to compare the expression of microglial markers in hiPSC-derived microglia with hiPSC-derived macrophages differentiated for the same number of days in vitro. This demonstrated high percentages (∼90%) of the macrophage/microglia marker, IBA1, in both hiPSC-derived macrophages and microglia (Fig. 1E). Meanwhile, hiPSC-derived microglia showed higher percentages of positive cells for the microglia enriched markers P2RY12 (Wilcoxon *p* = 0.001) and TMEM119 (*p* = 0.02) compared to hiPSC-derived macrophages (Fig. 1F-G). No significant differences in IBA1, P2RY12, or TMEM119 immunofluorescence were found between healthy control and *VCP* mutant samples within either cell type. Morphological analysis revealed that hiPSC-derived microglia cultures exhibited a greater proportion of more ramified features compared to hiPSC-derived macrophages. No significant differences in morphology were found between healthy control and *VCP* lines within either cell type (Fig. S1D). These findings confirm that our hiPSC-derived microglia express bona fide microglial markers and display microglial morphological characteristics.

### *VCP* mutant microglia display transcriptomic, proteomic and functional perturbations in lysosomal function and immune response

**Fig. 2:**
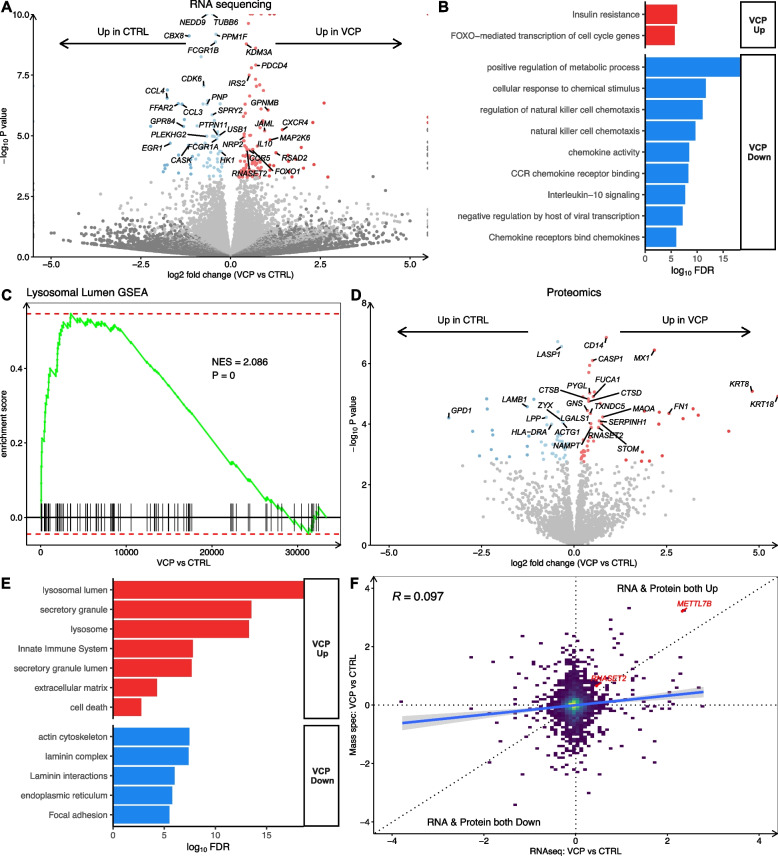
*VCP* mutant microglia display transcriptomic and proteomic perturbations. **A** Volcano plot showing log_2_ fold change in *VCP* mutant versus healthy control microglia differential gene expression. Genes with significantly (FDR < 0.05) increased expression are shown in red, and those decreased in expression are shown in blue. Data are from 3–4 lines per condition from 1 differentiation. **B** Gene Ontology (GO) terms enriched in upregulated (red) and downregulated (blue) differentially expressed genes in *VCP* mutant microglia. **C** Gene set enrichment analysis for lysosomal lumen genes in *VCP* mutant microglia. **D** Volcano plot showing log_2_ fold change in *VCP* mutant versus healthy control microglia differential protein abundance from mass spectrometry. Data are from 5 lines per condition from 1 differentiation. **E** GO terms enriched in upregulated (red) and downregulated (blue) differentially expressed proteins in *VCP* mutant microglia. **F** Correlation of VCP vs CTRL microglia in RNAseq (x-axis) with mass spectrometry (y-axis)

We next compared gene expression in *VCP* mutant versus healthy control hiPSC-derived microglia and found 183 differentially expressed genes (FDR < 0.05), with 110 upregulated and 73 downregulated in *VCP* mutant microglia, adjusted for isogenic sample relatedness (Fig. 2A, Table S3). Amongst these were the ALS-related genes, *PFN1* and *MAP2K6*, as well as genes encoding 10 RNA binding proteins (e.g. *PDCD4*, *USB1*, *ELAVL4*, *GNL1*, *EIF4B*). To further ensure these findings were not technical artefacts of sample relatedness between isogenic counterparts, we performed a sensitivity analysis excluding isogenic samples. This confirmed a strong positive correlation (Pearson’s *R* =  + 0.7) as well as significant overlap of differentially expressed genes (*n* = 42, 23%, Fisher Exact test *p* = 4.3 × 10^–60^) between the analysis without isogenic pairs and that including all samples (Fig. S2A-B).

Functional enrichment analysis revealed that upregulated genes were involved in PI3K binding (e.g. *PIK3IP1, IRS2, ATP1A1*), insulin resistance and FOXO signalling genes while downregulated genes were overrepresented by metabolic and immune processes (Fig. 2B). Gene set enrichment analysis (GSEA) revealed changes in several gene sets including an increase in lysosomal lumen (*n* = 91, normalised enrichment score [NES] + 2.1, *p *= 9.4 × 10^–7^; Fig. 2C), positive regulation of autophagy (*n* = 133, NES [normalised enrichment score] + 1.9, *p* = 1.07 × 10^–4^), and activation of immune response (*n *= 301, NES + 1.44, *p* = 0.017) gene sets, and a decrease in somatic diversification of immunoglobulins involved in the immune response (*n *= 50, NES -1.67, *p* = 0.038), respiratory electron transport chain (*n* = 112, NES -2.03, *p* = 2.11 × 10^–5^), DNA binding (*n* = 459, NES -1.49, *p* = 9.51 × 10^–4^) and RNA splicing (*n *= 411, NES -1.9, *p* = 1.26 × 10^–8^) gene sets (Table S4).

To further understand how signalling pathways are activated in *VCP* mutant microglia we performed a Pathway RespOnsive GENes (PROGENy) analysis[46]. *VCP* mutant microglia displayed activation of hypoxia (NES + 4.88, *p* = 0.002), JAK-STAT (NES + 4.53, *p* = 0.002), p53 (NES + 2.73, *p* = 0.006) and Wnt (NES + 2.39, *p *= 0.04) signalling and downregulation of MAPK (NES -11.12, *p* = 0.002), EGFR (NES -9.46,* p* = 0.002), PI3K (NES -6.93, *p* = 0.002), NFкβ (NES -3.8, *p *= 0.002) and Estrogen (NES -2.76, *p* = 0.012) signalling (Fig. S2C). We also inferred changes in the activity of transcription factors in *VCP* mutant microglia using Discriminant Regulon Expression Analysis (DoRothEA)[47] and found increased activity of FOXO3, FOXO1, ARNTL, and TP53 and decreased activity of E2F4, E2F1, and MYC (Fig. S2D).

We next examined changes in the proteome of *VCP* mutant microglia using shotgun data-dependent analysis (DDA) mass spectrometry. We identified 101 significant differentially expressed proteins (FDR < 0.05), of which 51 were upregulated and 50 downregulated in *VCP* mutant microglia compared to healthy control (Fig. 2D, Table S5). Functional enrichment analysis revealed upregulated proteins were related to lysosome (e.g. CTSB, FUCA1, CTSD, GNS and TXNDC5), secretion (e.g. CD14, PYGL, GNS), and immune activation (e.g. CASP1; Fig. 2E). Conversely, downregulated proteins were overrepresented by cytoskeletal functions, such as actin (e.g. FLNA and ACTG1) and laminin (LAMA1, LAMB1 and LAMC1) terms. Comparing *VCP* mutant microglia changes in mass spectrometry with RNAseq, revealed that although the global correlation was modest (*R* =  + 0.097), both METTL7B and RNASET2 were significantly increased in their transcript and protein in *VCP* mutant microglia (Fig. 2F).

As altered phagocytosis has been described in ALS microglia[30, 31] we further investigated phagocytosis in *VCP* mutant microglia. We observed a decrease in the phagocytosis gene set in *VCP* microglia (*n *= 265, NES -1.4, *p* = 0.01; Fig. S2E). To functionally assess phagocytosis, we measured uptake of pHrodo fluorescent bioparticles, however, we did not identify any significant differences between *VCP* mutant and healthy control microglia (Fig. S2F-G). Since metabolic processes were among downregulated terms in the enrichment analysis of transcriptomic data from *VCP* mutant microglia, we explored changes in mitochondria. We found a decrease in the mitochondrial gene set in *VCP* microglia (*n* = 181, NES -2.2, *p* = 1 × 10^–10^; Fig. S2H). Nevertheless, when functionally assessing mitochondrial membrane potential using tetramethylrhodamine (TMRM), we observed no significant differences between *VCP* mutant and healthy control microglia in TMRM signal intensity or mitochondrial mass (Fig. S2I-K).

**Fig. 3:**
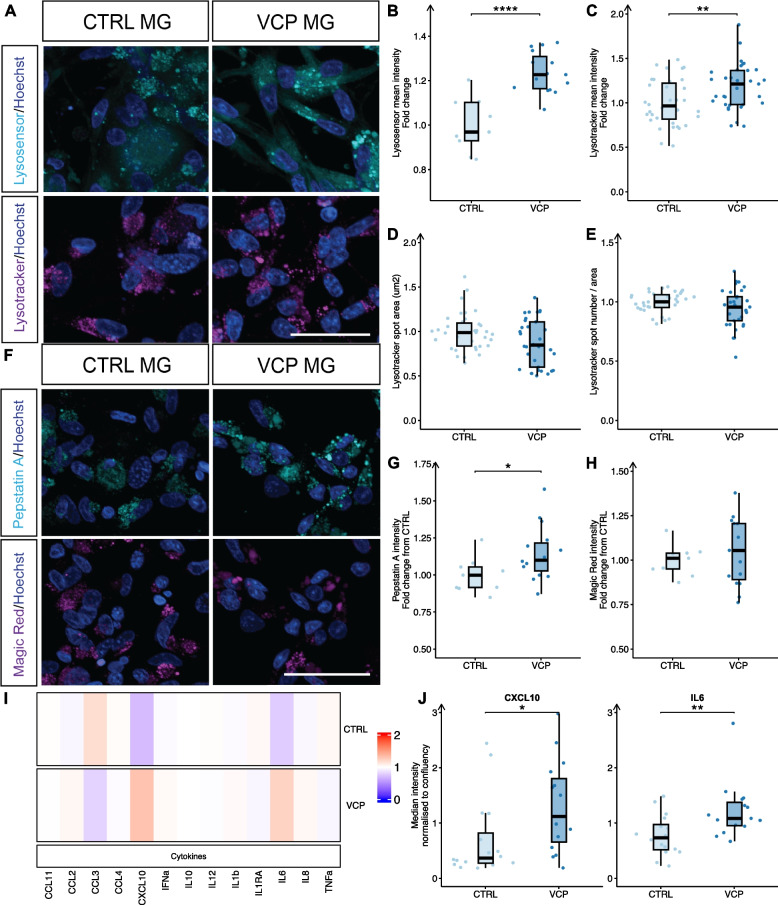
*VCP* mutant microglia display lysosomal and immune functional perturbations. **A** Representative images and quantification of mean Lysosensor (**B**), Lysotracker intensity (**C**), Lysotracker spot area (**D**) and Lysotracker spot number (**E**) measurements in CTRL and *VCP* mutant microglia. Scale bar: 50 μm. Data are from 3 experimental blocks from 3–4 lines per condition from 1–3 differentiations. **F** Representative images and quantification of cathepsin D (**G**) and cathepsin B (**H**) activity. Scale bar: 50 μm. Data are from 3 experimental blocks from 3–5 lines per condition from 1 differentiation. **I** Heatmap showing log_2_ fold change in differential basal secretion of cytokines and chemokines in *VCP* mutant microglia and median fluorescence intensity (MFI) plots for significantly changed and (**J**) IL-6 and CXCL10 secreted proteins. All stats are from the Wilcoxon test ** *p* < 0.01, * *p* < 0.05. Data points are individual cell lines (2–5 lines per condition from 2 differentiations, average of 2–3 technical repeats) from 4 experimental blocks

To further investigate changes in lysosomal function in *VCP* mutant microglia, as implicated by both transcriptomic and proteomic analyses, we conducted live cell imaging of Lysosensor and Lysotracker fluorescent dyes (Fig. 3A). In *VCP* mutant microglia, we identified increased intensity of Lysosensor signal (*p* = 2.9 × 10^–5^), increased intensity in Lysotracker signal (*p* = 0.007), and nonsignificant decreases in lysosomal area (*p* = 0.1) and the number of lysosomes per cell area (*p* = 0.1; Fig. 3B-E). These changes in Lysotracker and Lysosensor measurements were not observed in *VCP* mutant hiPSCs or microglia precursors (pMG) (Fig. S3). In addition, we measured activity of cathepsin D and cathepsin B using BODIPY FL-pepstatin A and Magic Red cathepsin B substrates, respectively. We observed an increase in cathepsin D (*p* = 0.04), but no change in cathepsin B activity (*p* = 0.5), (Fig. 3F-H). Noting that cathepsin D is more active at a lower pH [48, 49]*,* these data are consistent with a lower lysosomal pH in *VCP* mutant microglia.

Since dysregulation of immune signalling was also found at both transcriptomic and proteomic levels, we investigated the secretion of cytokines and chemokines in media from *VCP* mutant microglia. We detected basal levels of 13 out of 25 measured cytokines and chemokines and found that IL-6 (*p* = 0.02) and CXCL10 (*p* = 0.03) were significantly increased in *VCP* mutant microglia (Fig. 3I-J). In summary, our findings demonstrate that *VCP* mutant microglia not only exhibit transcriptomic and proteomic changes related to immune and lysosomal signalling but also display functional dysregulation in these processes.

### *VCP* mutant microglia show partial overlap in transcriptomic and proteomic signatures with LPS-stimulated microglia

**Fig. 4:**
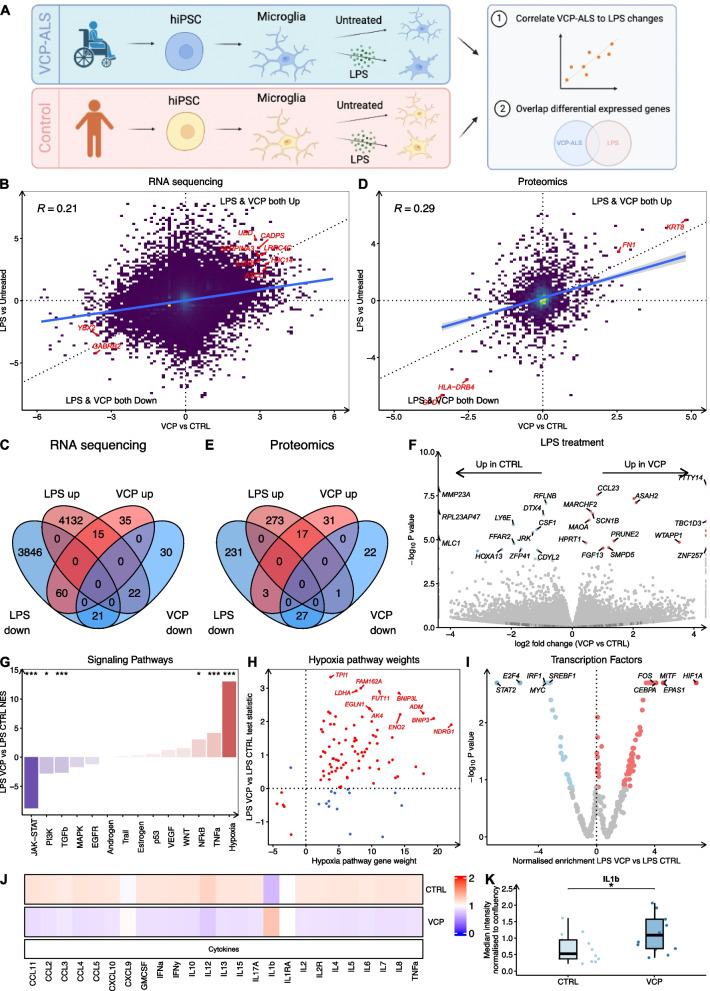
*VCP* mutant microglia show partial overlap with LPS-stimulated healthy control microglia. **A** Schematic showing treatment of healthy control and *VCP* mutant microglia with LPS and downstream RNA-seq and mass spectrometry analysis and comparison. **B** Scatterplot showing differential gene expression z-statistical (Wald test) between *VCP* mutant (x-axis) and LPS related (y-axis) changes. Data are from 3–4 lines per condition from 1 differentiation. **C** Venn diagram depicting the number of overlapping differentially expressed genes (FDR < 0.05) between *VCP* mutant and LPS-stimulated microglia. **D** Scatterplot showing differential protein expression z-statistical (Wald test) between *VCP* mutant (x-axis) and LPS related (y-axis) changes. Data are from 5 lines per condition from 1 differentiation. **E** Venn diagram depicting the number of overlapping differentially expressed proteins (FDR < 0.05) between *VCP* mutant and LPS-stimulated microglia. **F** Volcano plot of log_2_ fold change in differential gene expression between LPS-stimulated *VCP* mutant versus LPS-stimulated healthy control microglia. **G**, **H** PROGENy signaling pathway activity normalized enrichment scores (*y*-axis) in LPS-stimulated *VCP* mutant versus LPS-stimulated healthy control microglia. **I** DoRothEA transcription factor regulon analysis in *VCP* mutant microglia. **J** Heatmap showing differentially secreted cytokines and chemokines in LPS-stimulated *VCP* mutant versus LPS-stimulated healthy control microglia. **K** Median fluorescence intensity (MFI) plot for IL-1B release in LPS-stimulated *VCP* mutant versus LPS-stimulated healthy control microglia

Since *VCP* mutant microglia demonstrated perturbations in inflammation, we next addressed whether *VCP* mutations induce a similar reactive state in microglia to that of LPS-stimulated healthy control counterparts (Fig. 4A). PCA demonstrated substantial separation between unstimulated and LPS-stimulated microglia in PC1 (Fig. S4A) and we detected large numbers of gene expression changes, with 4,169 upregulated and 3,928 downregulated differentially expressed genes (FDR < 0.05, Fig. S4B, Table S6). Functional enrichment analysis revealed that upregulated genes were enriched in inflammatory terms, while downregulated genes were involved in mitochondrial functions and mRNA translation (Fig. S4C). Signalling pathway analysis identified that LPS stimulation led to profound upregulation of NFкβ (NES + 23.6, *p* < 0.002), TNFa (NES + 20.9, *p* < 0.002), JAK-STAT (NES + 16.1, *p* < 0.002) and hypoxia (NES + 9.1, *p* < 0.002) signalling (Fig. S4D). Transcription factor activity analysis showed that LPS-stimulated microglia upregulated RELA, NFкβ1, STAT2, STAT1, RELB, and HIF1A (Fig. S4E).

Using mass spectrometry, we identified 291 upregulated and 261 downregulated differentially expressed proteins in LPS stimulated versus unstimulated microglia (Fig. S4F, Table S7). Functionally enriched terms overrepresented amongst upregulated proteins included extracellular vesicles, cytokine signalling and immune/stress response, whilst downregulated proteins were enriched for lysosome and exocytosis terms (Fig. S4G). Examining microglia conditioned media found increased secretion of almost all 25 cytokines and chemokines measured in LPS-stimulated microglia, with the greatest increases in IL-10, IL-12 and TNFa (Fig. S4H).

To assess whether *VCP* mutant microglia shared gene expression changes with LPS-stimulated microglia, we correlated *VCP* mutant and LPS-stimulated gene expression changes. This revealed a moderate positive association (*R* =  + 0.21; Fig. 4B), consistent with shared transcriptome-wide alterations between *VCP* mutant and LPS-stimulated microglia. Of the 110 genes significantly upregulated in *VCP* microglia, 15 (13.6%) were also significantly upregulated in LPS-stimulated microglia (Fisher’s exact test *p *= 0.008; Fig. 4C). Similarly, of the 73 genes significantly downregulated in *VCP* microglia, 21 (28.8%) were also downregulated in LPS-stimulated microglia (*p* = 4.2 × 10^–9^; Table S8). However, we also found genes changing in opposing directions with 60 genes increased in *VCP* mutant being decreased with LPS-stimulation and 22 genes decreased in *VCP* mutant being increased with LPS. At the protein level, a similar positive association was observed between *VCP* mutant and LPS stimulated changes (*R* =  + 0.29, Fig. 4D), with 17 shared upregulated and 27 co-downregulated proteins (Fig. 4E). Thus, although the global correlations are modest, *VCP* mutant and LPS-stimulated hiPSC microglia share some reactive changes.

### *VCP* mutant microglia show augmented activation of hypoxia and inflammatory signalling in response to LPS

We next sought to compare differences in the response to LPS between *VCP* mutant microglia and healthy control microglia. Comparing gene expression in LPS-stimulated *VCP* mutant microglia versus LPS-stimulated healthy control microglia revealed 30 differentially expressed genes of which 16 were upregulated and 14 downregulated in LPS-stimulated *VCP* mutant microglia (Fig. 4F). Signalling pathway analysis showed that LPS-stimulated *VCP* mutant microglia displayed augmented activation of hypoxia (NES + 13.4, *p* < 0.001), TNFα (NES + 3.42, *p* < 0.001), and NFкβ (NES + 3.0, *p* = 0.01) signalling, whilst they displayed diminished activation of JAK-STAT (NES -14.6, *p* < 0.002), PI3K (NES -2.8, *p* = 0.016), and TGFβ (NES -2.7, *p* = 0.008) pathways (Fig. 4G-H). Analysis of transcription factor regulon activity showed that HIF1A had the most increased activity in LPS-stimulated *VCP* mutants, consistent with hypoxia-related signalling, whilst STAT2 showed the most decreased activity (Fig. 4I). Furthermore, analysis of microglia media revealed increased secretion of pro-inflammatory cytokine IL-1β (*p* = 0.03) in LPS-stimulated *VCP* mutant microglia compared with LPS-stimulated healthy control microglia (Fig. 4J-K). These findings indicate that *VCP* mutant microglia exhibit distinct differences in their response to LPS stimulation, characterised by dysregulated hypoxia and inflammatory signalling at the transcriptomic level and increased secretion of IL-1β.

### *VCP* mutant microglia share gene expression changes with a *SOD1* mutant mouse model and a large human post-mortem ALS spinal cord database

**Fig. 5:**
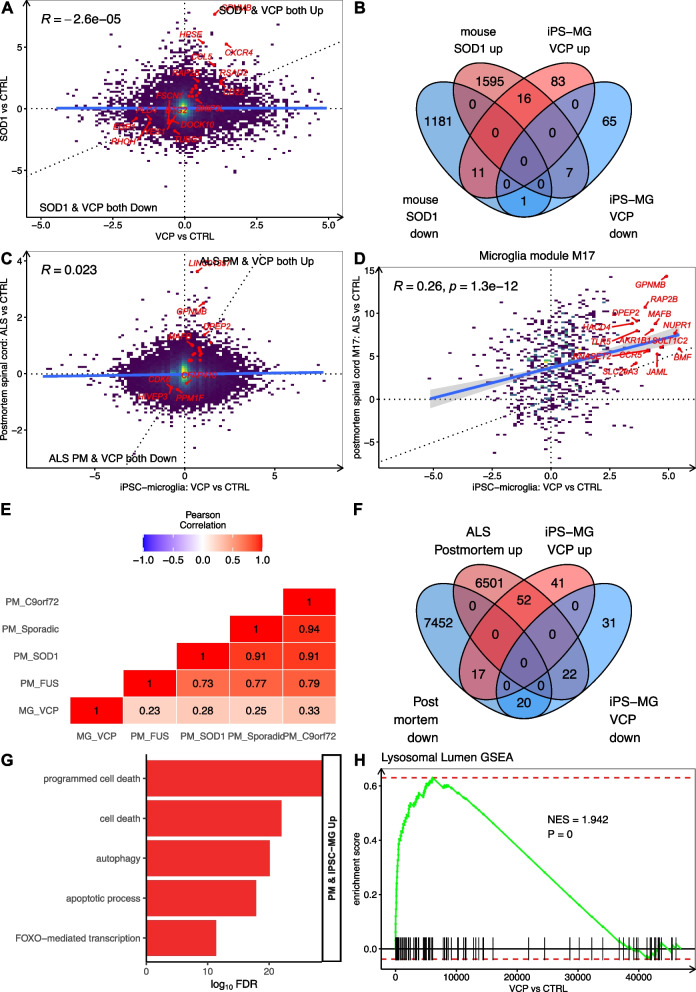
*VCP* mutant microglia share microglial gene expression changes with *SOD1* mutant mice and post-mortem ALS spinal cords. **A** Scatterplot of the differential gene expression z-statistical (Wald test) between *VCP* mutant microglia (x-axis) and microglia from *SOD1* mutant mice (y-axis). **B** Venn diagram showing overlap of differentially expressed genes (FDR < 0.05) between *VCP* mutant microglia and *SOD1* mutant mouse microglia. **C** Scatterplot of differential gene expression z-statistical (Wald test) between *VCP* mutant (x-axis) and post-mortem ALS spinal cord (y-axis). **D** Scatterplot of differential gene expression z-statistical (Wald test) between *VCP* mutant (x-axis) and microglia M17 module (y-axis) from post-mortem ALS spinal cord. **E** Heatmap showing the Pearson’s correlation coefficient for transcriptome-wide changes between each post mortem ALS genetic background and *VCP* mutant microglia. **F** Venn diagram of differentially expressed genes (FDR < 0.05) between *VCP* mutant microglia and post-mortem ALS spinal cord. **G** GO terms enriched in co-upregulated differentially expressed genes in *VCP* mutant microglia and post-mortem ALS spinal cord. **H** Gene set enrichment analysis for lysosomal lumen genes in ALS spinal cord

To assess whether the gene expression signatures of *VCP* mutant hiPSC-derived microglia are also found in other models of ALS, we compared our findings with microglia isolated from end-stage *SOD1* mutant mouse spinal cord [50]. While we found no global transcriptome-wide correlation between *VCP* and *SOD1* mutant gene expression changes (*R* = -0.04), possibly due to interspecies differences (Fig. 5A), there was a significant overlap between genes upregulated in *VCP* and *SOD1* mutant microglia, including *GPNMB, CXCR4, RASSF3, NRP2, CALM3, BNIP3L* (*n* = 16, 14.5%, Fisher’s exact test *p* = 1.4 × 10^–7^; Fig. 5B, Table S9). The sole co-downregulated gene was the DNA and RNA binding protein, *JRK*.

To examine whether changes in *VCP* mutant microglia are present in postmortem ALS spinal cord tissue we compared our hiPSC-derived *VCP* mutant microglia with the NYGC ALS Consortium database[22] consisting of 214 ALS patients and 57 healthy controls, which spans non-mutant cases (*n* = 161), *C9orf72* (*n* = 36), *SOD1* (*n* = 5), *FUS* (*n* = 2), and 8 other ALS mutations[51]. We compared global gene expression changes between *VCP* mutant microglia and all postmortem ALS spinal cord samples, irrespective of genetic status. This revealed limited correlation (*R* =  + 0.02, Fig. 5C), perhaps due to the multiple cell types present in spinal cord samples. Indeed, harnessing a gene co-expression network analysis from the same postmortem samples, we found that *VCP* mutant microglia showed stronger correlations with the microglial specific module (M17; *R* =  + 0.26, *p* = 1 × 10^–12^; Fig. 5D)[22]. To identify whether microglial gene expression changes in *VCP* mutant microglia align more closely with a particular genetic subgroup, we correlated changes with each genotype separately. This revealed marginally stronger correlations between *VCP* mutant microglia and *C9orf72* mutants (*R* =  + 0.33), followed by *SOD1* (*R* =  + 0.28), sporadic (*R* =  + 0.25) and *FUS* (*R* =  + 0.23) (Fig. 5E).

Intersecting differentially expressed genes between *VCP* microglia and postmortem ALS confirmed a significant overlap in both upregulated (52/110, 47%, Fisher *p* = 7 × 10^–22^) and downregulated (20/73, 31.5%, *p* = 4 × 10^–4^) genes (Fig. 5F, Table S10). Meanwhile, several significantly differentially expressed genes were also identified in opposing directions, including upregulated in *VCP* microglia but downregulated in postmortem ALS (17/110, 15.5%) and downregulated in *VCP* microglia but upregulated in postmortem ALS (22/73, 30%). Functional enrichment analysis of overlapping genes revealed increased expression of genes relating to cell death, autophagy and response to stress (Fig. 5G). As with *VCP* mutant microglia, GSEA revealed the lysosomal lumen gene set was strongly increased in post mortem ALS spinal cords (*n* = 91, NES + 2.1, *p* = 7.4 × 10^–9^; Fig. 5H). Overall, hiPSC-derived *VCP* microglia and postmortem ALS microglia share a common set of altered genes, particularly those associated with lysosomes. Notably, *GPNMB* was commonly upregulated in *VCP* mutant hiPSC-derived microglia, *SOD1* mutant mouse microglia, and post-mortem ALS spinal cords, and its increased protein expression has been reported in ALS spinal cord, CSF and serum[22, 52].

### GPNMB affects inflammatory transcriptomic responses and phagocytic processes independently of *VCP* mutant microglial phenotypes

**Fig. 6:**
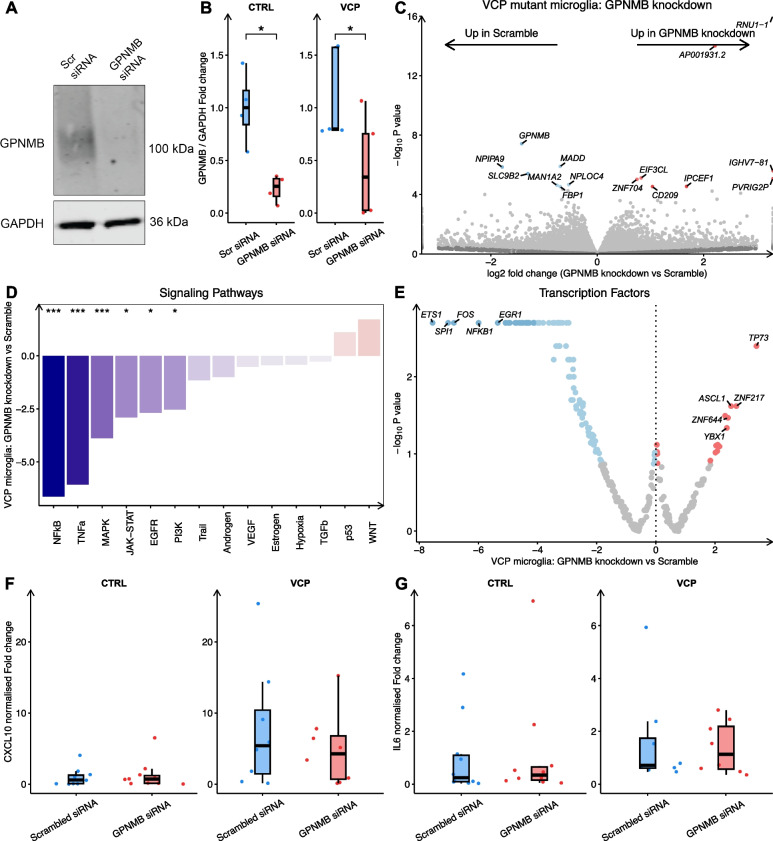
*GPNMB* knockdown reduces inflammatory signalling in *VCP* mutant microglia. **A** Western blot for GPNMB and GAPDH loading control in microglia treated with scrambled or GPNMB targeting siRNA. **B** Quantification of western blot for GPNMB in microglia treated with scrambled or GPNMB targeting siRNA. **C** Volcano plot of log_2_ fold change in differential gene expression between *VCP* mutant microglia treated with GPNMB siRNA or scrambled siRNA. **D** PROGENy signaling pathway activity normalized enrichment scores (*y*-axis) in GPNMB siRNA treated *VCP* mutant microglia. **E** DoRothEA transcription factor regulon analysis in GPNMB siRNA treated *VCP* mutant microglia. Data are from 3 lines per condition from 1 differentiation. Quantification of (**F**) CXCL10 and (**G**) IL-6 secretion from healthy control and *VCP* mutant microglia treated with scrambled or *GPNMB* siRNA. Statistics are from a generalised linear model accounting for experimental repeats. Data are from 4 lines per condition from 2 differentiations

GPNMB is a transmembrane glycoprotein implicated in a multitude of cellular processes including proliferation, cell adhesion and inflammation and has been reported to be increased in expression in several microglial reactive states including in Alzheimer’s disease[53, 54], Parkinson’s disease[55] and diabetic retinopathy[56]. We next explored the role of GPNMB in hiPSC-derived *VCP* mutant microglia. We performed siRNA mediated knockdown of *GPNMB* in healthy control and *VCP* mutant microglia, confirming knockdown by qPCR, RNAseq and Western blot (Fig. 6A-B; Fig. S5A-B). We found no differences in the localisation of GPNMB in *VCP* mutant microglia (Fig. S5D-G). We next performed qPCR to examine *GPNMB* expression at various stages of microglia differentiation. This revealed negligible *GPNMB* expression in hiPSCs, a modest increase in expression in microglia precursors, and higher expression in differentiated microglia (Fig. S5C). These findings indicate a progressive upregulation of *GPNMB* as cells transition towards a microglial state.

Using RNA-seq, we first investigated the role of *GPNMB* in healthy control microglia. Comparing microglia treated with *GPNMB* siRNA to scrambled non-targeting siRNA revealed 19 differentially expressed genes with 6 upregulated and 13 downregulated genes (Fig. S5H; Table S11). GSEA revealed increases in extracellular matrix, insulin-like growth factor signalling, differentiation and angiogenesis gene sets, while downregulated gene sets included immune and DNA damage responses, ubiquitination, autophagy, protein localisation, lipid metabolism and oxidative phosphorylation (Table S12). Signalling pathway analysis revealed decreased JAK-STAT (NES -13, *p* < 0.002), TNFα (NES -4.29, *p* < 0.002), NFкβ (NES -4.29 *p* < 0.002) and MAPK (NES -3, *p* = 0.012) signalling (Fig. S5I). Transcription factor activity analysis revealed increased activity in HOXB13, DEAF1, SNAI1 and decreased activity in STAT2, NFкβ1, SPI1, RELA and IRF1 (Fig. S5J). siRNA mediated knockdown of *GPNMB* also resulted in a functional decrease in phagocytosis, measured by uptake of pHrodo fluorescent bioparticles (Fig. S5K-L).

To investigate any potential ability of *GPNMB* knockdown in *VCP* mutant microglia to rescue disease phenotypes, we compared *VCP* mutant microglia treated with *GPNMB* siRNA to *VCP* mutant microglia treated with scrambled siRNA. We identified 15 differentially expressed genes, with 7 upregulated and 8 downregulated genes (Fig. 6C; Table S13). GSEA revealed increases in translation, and antigen presentation gene sets and decreases in immune responses and mitochondria gene sets in *GPNMB* siRNA-treated *VCP* mutant microglia (Table S14). Signalling pathway analysis revealed that *GPNMB* siRNA-stimulated *VCP* mutant microglia displayed downregulation of NFкβ (NES -6.63, *p* < 0.002), TNFα (NES -6.07,* p *< 0.002), MAPK (-3.87, *p* < 0.002), JAK-STAT (NES -2.71, *p *= 0.016), EGFR (NES -2.69, *p *= 0.018) and PI3K (NES -2.53, *p* = 0.018) signalling (Fig. 6D). Furthermore, transcription factor activity analysis found TP73 among transcription factors with increased activity and decreased activity of ETS1, SPI1, FOS and NFкβ (Fig. 6E). We next sought to examine whether *GPNMB* knockdown would reverse functional phenotypes in *VCP* mutant microglia. However, *GPNMB* knockdown did not reduce increased CXCL10 or IL-6 secretion from *VCP* mutant microglia (Fig. 6F-G). Furthermore, *GPNMB* knockdown did not affect lysosomal function when measuring lysosensor intensity, lysotracker intensity, spot size or number or cathepsin B and cathepsin D activity in *VCP* mutant microglia (Fig. S6).

### *VCP* mutant and LPS stimulated microglia share non cell autonomous effects of JAK-STAT activation in motor neurons and astrocytes

**Fig. 7:**
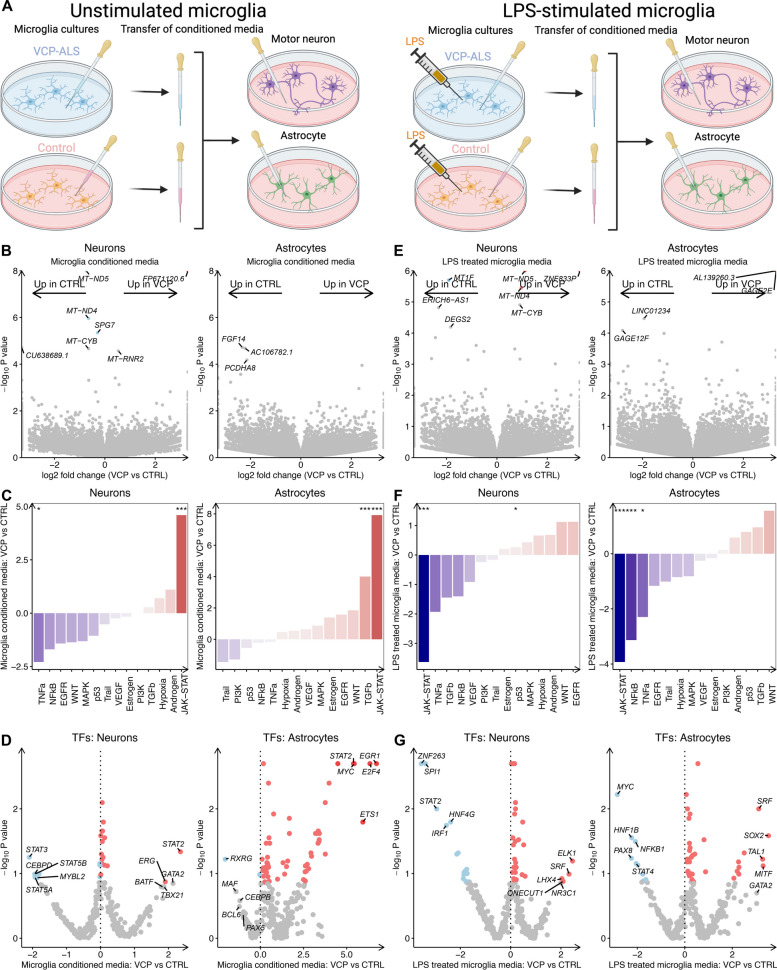
*VCP* mutant and LPS-stimulated microglia conditioned media activates JAK-STAT signalling in motor neurons and astrocytes. **A** Schematic of microglia conditioned media transfer in unstimulated and LPS-stimulated microglia. **B** Volcano plot of log_2_ fold change in differential gene expression between *VCP* mutant microglia conditioned media and healthy control microglia conditioned media on hiPSC derived healthy control motor neurons and astrocytes. **C** PROGENy signaling pathway activity normalized enrichment scores (*y*-axis) in unstimulated *VCP* mutant microglia conditioned treated healthy control neurons and astrocytes. **D** DoRothEA transcription factor regulon analysis in unstimulated *VCP* mutant microglia conditioned treated healthy control neurons and astrocytes. **E** Volcano plot of log_2_ fold change in differential gene expression between LPS-stimulated *VCP* mutant microglia conditioned media vs LPS-stimulated healthy control microglia conditioned media treated healthy control neurons and astrocytes. **F** PROGENy signaling pathway activity normalized enrichment scores (*y*-axis) in LPS-stimulated *VCP* mutant microglia conditioned media vs LPS-stimulated healthy control microglia conditioned media treated healthy control neurons and astrocytes. **G** DoRothEA transcription factor regulon analysis in LPS-stimulated *VCP* mutant microglia conditioned media vs LPS-stimulated healthy control microglia conditioned media treated healthy control neurons and astrocytes. Data are from 3 healthy control lines per condition from 1 differentiation for both motor neurons and astrocytes

To investigate whether the secretome of *VCP* mutant microglia induces changes in motor neurons and astrocytes, we incubated hiPSC-derived motor neurons and astrocytes with microglia conditioned media for 72 h at a ratio of 50:50 with fresh media, using our previously published protocols to obtain motor neurons and astrocytes (Fig. 7A)[34, 43]. Using a caspase-3 based longitudinal live imaging platform we found no significant differences in survival between motor neurons treated with conditioned media from healthy control or *VCP* mutant microglia (Fig. S7A-B). Transcriptomic analysis of conditioned-media treated motor neurons from *VCP* mutant compared with healthy control microglia revealed only 4 differentially expressed genes while there were no significant differentially expressed genes in conditioned-media treated astrocytes (Fig. 7B; Table S15-16). Gene Ontology revealed that downregulated genes in motor neurons (*SPG7*, *MT-ND4* and *MT-ND5*) were enriched in mitochondrial functions. GSEA found that in motor neurons increases in translation and decreases in DNA packaging and toll-like receptor 4 regulation gene sets. In astrocytes, GSEA revealed increases in translation, antiviral signalling and mitochondria gene sets and decreases in ion channel activity (Table S17-18). Signalling pathway analysis revealed that *VCP* mutant microglia conditioned media upregulated JAK-STAT signalling in both motor neurons and astrocytes and analysis of transcription factor activity revealed that STAT2 activity was increased in both cell types (Fig. 7C-D).

We next sought to further understand the non cell autonomous roles of microglial GPNMB. We performed *GPNMB* knockdown in healthy control and *VCP* mutant microglia and examined the non cell autonomous effect of conditioned media on survival of healthy control and *VCP* mutant motor neurons under basal conditions and after treatment with proteasome inhibitor, MG132. Although MG132 elicited motor neuron death, we found no significant differences in motor neuron survival in both control and *VCP* mutant motor neurons after treatment with conditioned media from *GPNMB* knockdown microglia (Fig. S8). Furthermore, we did not observe any significant differences in motor neuron survival in both control and *VCP* mutant motor neurons physically co-cultured with control and *VCP* mutant microglia after *GPNMB* knockdown (Fig. S9). Taken together, microglial *GPNMB* knockdown does not impair motor neuron survival.

Since *VCP* mutant microglia shared transcriptomic and proteomic changes with LPS-stimulated microglia, we next sought to understand the impact of LPS stimulation on the contact-independent effects of microglia on astrocytes and neurons. In order to address this, we investigated motor neuron and astrocyte responses to i) the direct effects of fresh media containing LPS and ii) conditioned media from LPS-stimulated microglia. Motor neurons and astrocytes directly treated with fresh media containing LPS showed no significant differentially expressed genes, with only minimal changes in signalling pathway and transcription factor activity, indicative of modest direct effects of LPS (Fig. S7C-F; Table S19-21). Although media from LPS-stimulated healthy control microglia resulted in no significant gene expression changes in motor neurons compared to unstimulated healthy control microglia, it led to 11 differentially expressed genes in astrocytes, with upregulated genes involved in inflammatory processes (*VCAM1, C3, IFI6*; Fig. S7G-H; Table S22-25). Signalling pathway analysis revealed significantly increased JAK-STAT, TNFα and NFкβ signalling and increased activation of inflammatory transcription factors including STAT2 in both motor neurons and astrocytes (Fig. S7I-L). These findings confirm that the observed changes in motor neurons and astrocytes are primarily attributed to the secretome of LPS-stimulated microglia, rather than the direct effects of LPS itself. Furthermore, we discovered similarities in the responses of motor neurons and astrocytes to the secretomes of *VCP* mutant microglia and LPS stimulated microglia, characterised by activation of the JAK-STAT pathway and inflammatory responses.

Finally, we sought to investigate the differences in the responses of motor neurons and astrocytes to conditioned media from LPS-stimulated *VCP* mutant and LPS-stimulated healthy control microglia treated (Fig. 7A). In motor neurons, we identified three genes that were significantly upregulated (*ZNF833P*, *MT-ND4* and *MT-ND5)* and only *MTF1* that was downregulated upon exposure to conditioned media from LPS stimulated *VCP* mutant microglia compared to LPS-stimulated healthy control microglia conditioned media (Table S26). GSEA revealed that the *VCP* mutant microglia secretome upregulated the mitochondrial electron transport chain and downregulated the responses to heavy metals in motor neurons (Table S27). In astrocytes, the antigen encoding gene *GAGE2E* was significantly increased upon exposure to media from LPS-stimulated *VCP* mutant microglia compared to LPS-stimulated healthy control microglia (Fig. 7E; Table S28). GSEA revealed that *VCP* mutant LPS-stimulated microglia media led to downregulation of developmental, metabolic and synaptic functions in astrocytes compared with astrocytes treated with healthy control microglia stimulated with LPS (Table S29). Conversely to what was observed for the effects of unstimulated *VCP* mutant microglia, the LPS-stimulated *VCP* mutant microglial secretome led to a profound and significant decrease in JAK-STAT signalling in both motor neurons (NES -3.59, *p* = 0.002) and astrocytes (NES -3.87, *p* = 0.002; Fig. 7F-G). These data collectively suggest that the *VCP* mutant microglia secretome induces JAK-STAT activation in motor neurons and astrocytes; however upon LPS-stimulation *VCP* mutant microglia show a blunted ability to activate JAK-STAT.

## Discussion

Exploring the impact of microglia in ALS is crucial for a comprehensive understanding of disease pathomechanisms. Here, we extensively studied the transcriptomic, proteomic and functional alterations in hiPSC-derived *VCP* mutant ALS microglia, as well as the effects of their secretome on motor neurons and astrocytes. We identified multiomic and functional changes relating to lysosomal and immune dysfunction. Changes in *VCP* mutant microglia displayed similarities with LPS-stimulated microglia across transcriptomic and proteomic signatures. *VCP* mutant microglia also displayed augmented inflammatory activation following LPS stimulation compared with healthy control microglia. Importantly, we identified a core set of genes that were consistently altered in *VCP* mutant microglia, postmortem ALS spinal cord, and *SOD1* mutant mice, which may represent attractive therapeutic targets to modulate microglial dysfunction in ALS. While knockdown of *GPNMB* reduced inflammatory processes and phagocytosis in microglia, it did not affect *VCP* mutant phenotypes or rescue motor neuron death under conditions of stress, suggesting that *GPNMB* does not directly affect *VCP* related phenotypes.

The advent of protocols to derive microglia from hiPSCs has facilitated the study of ALS patient derived cells in vitro. *FUS* mutant hiPSC derived microglia were recently shown to elicit a reactive transcriptomic signature but found minimal effects on secreted factor release [33]. Meanwhile, *C9orf72* mutant hiPSC-derived microglia underwent modest transcriptional changes but displayed endosomal-lysosomal dysfunction and altered cytokine release profiles, albeit only in response to LPS [30–32]. Our study of *VCP* mutant hiPSC-derived microglia also found increased IL-1β release after LPS stimulation, but not TNF-α or IL-6 as reported in these studies. Furthermore, we identified changes at the transcriptomic and proteomic levels and found basal differences in the secretion of IL-6 and CXCL10 in *VCP* mutant hiPSC-derived microglia.

In both hiPSC-derived *VCP* mutant microglia and microglia from postmortem ALS spinal cords, we identified upregulation of the lysosomal lumen gene set as well as an overlapping set of upregulated lysosomal genes. Some of these lysosomal genes have also been found to be elevated in disease associated microglia (DAM) in *SOD1* and *APP* mutant mouse models [50, 57]. Lysosome function is increasingly recognised to extend beyond degradation of cellular waste, and is now recognised to play important roles in immune processes, plasma membrane repair and metabolism [58–60]. VCP has been shown to play an important role in the clearance of damaged lysosomes [4]. Dysfunction of lysosomes has been linked with several neurodegenerative diseases through proteotoxic stress, immune and metabolic dysregulation [61, 62]. In addition, during normal ageing mouse microglia have been reported to undergo lysosomal dysfunction and lose their spatial organisation in the ventral horn of the spinal cord, clustering around motor neurons [63]. Lysosomes hold potential as an attractive target for therapeutic intervention in ALS, as in addition to *VCP*, several other ALS associated genes have a role in lysosomal homeostasis (*C9orf72*, *TARDBP*, *TMEM106B*, *TBK1*, *OPTN, SQSTM1*) and several lysosomal storage diseases manifest neurological features [62, 64].

We observed dysregulated immune signalling in *VCP* mutant microglia at the gene expression level, particularly heightened PI3K and FOXO3 activity, which has previously been reported with microglial responses to inflammatory stimuli [65–67]. Furthermore, we identified increased secretion of IL-6 and CXCL10 from *VCP* mutant microglia suggesting functional dysregulation of immune signalling. Increased IL-6 gene expression has previously been reported in *C9orf72* knockout mouse spinal cord microglia, coinciding with an increased accumulation of lysosomes [68]. IL-6 has been reported to be elevated in ALS patients’ cerebrospinal fluid [69], serum [70] and plasma [71, 72]. IL-6 expression negatively correlates with disease duration [70] and functional scores (ALSFRS-R and MMT) [71], however another study reported no correlation of IL-6 levels with the ALSFRS-R [73]. Conversely, although elevated CXCL10 has also been reported in the cerebrospinal fluid of ALS patients it negatively correlates with disease progression. Further work is required to understand the contribution of IL-6 and CXCL10 to ALS.

We identified changes in lysosomal and immune pathways across transcriptomic, proteomic and functional levels in *VCP* mutant microglia. However, for certain phenotypes, including immune, mitochondrial and phagocytosis, there was discordance between transcriptomics, proteomics and functional assays. This may be due to the non-linear relationship between transcription and translation, which underscores the importance of multimodal evaluation when validating functional consequences of transcriptomic changes. Possible reasons for a lack of concordance between these datasets include post-transcriptional and post-translational regulation mechanisms, differences in the half lives of mRNA and proteins and technical differences in these methodologies e.g. proteomics can only identify a small fraction of the entire proteome and is currently less sensitive than RNAseq. A vast number of studies, not just within microglial activation, have reported discordance between RNAseq and mass spectrometry results [74–76].

We found that *GPNMB* expression was increased across hiPSC-derived *VCP* mutant microglia, *SOD1* mutant mouse microglia, and postmortem ALS spinal cords. GPNMB has been implicated in many cellular processes including proliferation, cell adhesion and inflammation and shows increased expression in several microglial reactive states including in Alzheimer’s disease [53, 54], Parkinson’s disease [55] and diabetic retinopathy [56]. Furthermore, a variant in *GPNMB* has been linked to Parkinson’s disease [77, 78]. GPNMB has been proposed as a biomarker in ALS where an increase in GPNMB in cerebrospinal fluid correlates with shorter survival [52]. Knockdown of *GPNMB* in healthy control microglia suggested that GPNMB is involved in several disease relevant pathways in microglia including immune and DNA damage responses, ubiquitination, autophagy, and lipid metabolism. Furthermore, we found decreased phagocytosis in *GPNMB* knockdown control human microglia. However, we found no effect of microglial *GPNMB* knockdown on *VCP* mutant microglial phenotypes or on motor neuron survival.

Conflicting evidence has been reported regarding the inflammatory and protective roles of GPNMB [39]. In agreement with our findings, knockdown of *GPNMB* in BV2 cells reduced TNF-α, IL-1β and nitric oxide production following LPS stimulation [79]. This is contrary to previously reported anti-inflammatory roles of GPNMB, where knockdown of *GPNMB* in mouse bone marrow derived monocytes exacerbated TNF-α, IL-1β and iNOS and expression following stimulation with LPS and IFN-γ [80]. Furthermore, the extracellular fragment of GPNMB partially attenuated induction of IL-6 gene expression in primary mouse astrocytes treated with TNFα, IL-1β and IFN-γ [81] and *GPNMB* overexpression reduced LPS induced IL-6 and NO production in RAW264.7 macrophages [82]*.* There is also evidence suggesting that GPNMB promotes neuroprotection as recombinant GPNMB partially rescued survival of NSC34 cells transfected with mutant SOD1 or TDP-43 and overexpression of GPNMB extended survival in *SOD1* mutant mice [83–85]. These studies may suggest cell type or context specific roles for GPNMB.

We found similarities between *VCP* mutant microglia and LPS-stimulated microglia. Interestingly, LPS and interferon-γ-stimulated hiPSC-derived microglia have also shown concordant changes with microglia from Alzheimer's disease post mortem tissue [86]. However, it should be noted that these correlations are relatively weak and several genes were differentially expressed in opposing directions, suggesting largely distinct activation states, which is consistent with the current consensus that microglial reactive states are highly context dependent [11].

Another key finding in our study was that LPS-stimulation augments hypoxia signalling in *VCP* mutant microglia. The impact of hypoxia signalling in ALS remains unclear, as it can have both detrimental or protective effects. While stabilisation of HIF1α, the master regulator of hypoxia signalling, has shown protective effects by extending lifespan in *SOD1* mutant mice[87], intermittent hypoxia exposure accelerated motor neuron loss [88]. Additionally, LPS-stimulation also amplifies NFкβ and TNF-α responses in *VCP* mutant microglia, both of which are implicated in ALS postmortem spinal cords and *SOD1* mutant mice [22, 27, 89]. Furthermore, diminished PI3K and TGFβ signalling may compromise potentially protective functions of microglia [90, 91]. These findings raise the possibility that cell autonomous changes in *VCP* mutant microglia may exacerbate harmful responses and diminish protective responses when responding to disease states.

We also found that the secretome from *VCP* mutant and LPS-stimulated microglia both induced profound JAK-STAT activation in motor neurons and astrocytes. Furthermore, the secretome from LPS-stimulated *VCP* mutant microglia intriguingly led to a blunted JAK-STAT activation in motor neurons and astrocytes. These findings suggest that the *VCP* mutation impairs the capacity of LPS-stimulated microglia to activate JAK-STAT signalling through a contact independent mechanism in neurons and astrocytes. In astrocytes, but not motor neurons, secreted factors from *VCP* mutant microglia also induced TGFβ signalling. Previous studies have shown that overexpression of TGFβ in astrocytes worsens disease in the mutant SOD1 mouse model of ALS and inhibiting TGFβ extended survival [92]. We have previously identified cell autonomous activation of TGFβ signalling in mutant ALS hiPSC astrocytes [35], suggesting that secreted factors from *VCP* mutant microglia are able to partially recapitulate ALS related signalling pathways in healthy control astrocytes.

Overall, our study provides insights into characterising the dysregulation of *VCP* mutant microglia. We identified multiomic and functional early cell autonomous perturbations in *VCP* mutant microglia in immune and lysosomal pathways, and their secretome elicited JAK-STAT activation in motor neurons and astrocytes. Further mechanistic dissection of these pathways will provide new understanding and possible therapeutic targets in ALS.

## Methods

### Compliance with ethical standards

For human iPSC work, informed consent was obtained from all patients and healthy controls in this study. Experimental protocols were all carried out according to approved regulations and guidelines by UCLH’s National Hospital for Neurology and Neurosurgery and UCL Queen Square Institute of Neurology joint research ethics committee (09/0272).

### hiPSC culture

Derivation of human fibroblasts and hiPSC dermal fibroblasts were cultured in OptiMEM + 10% FCS medium. The following episomal plasmids were transfected for hiPSC generation: pCXLE hOct4 shp53, pCXLE hSK, and pCXLE hUL (Addgene), as previously reported [93]. All cell cultures were maintained at 37 °C and 5% carbon dioxide. hiPSCs were maintained on Geltrex (Life Technologies) with Essential 8 Medium media (Life Technologies), and passaged using EDTA. Details of the lines used in this study are provided in supplementary Table 1.

### Differentiation of hiPSCs to macrophage and microglia-like cells

hiPSCs were differentiated into macrophages and microglia-like cells based on a previously defined protocol [38]. Briefly, hiPSCs were seeded into Aggrewell 800 wells to encourage embryoid body (EB) formation in E8 media supplemented with 50 ng/mL BMP4 (Peprotech), 50 ng/mL VEGF (Peprotech), and 20 ng/mL SCF (Miltenyi Biotec) to specify hemogenic endothelium. After at least 6 days, EBs were transferred to Geltrex coated flasks in factory media containing 100 ng/mL M-CSF (Invitrogen) and 25 ng/mL IL-3 (R&D), 2 mM Glutamax, penicillin/streptomycin and 0.055 mM β-mercaptoethanol in X-VIVO 15 media (Lonza). Fresh media was added weekly. Macrophage precursors were harvested from the supernatant from 5 weeks in vitro. Harvested cells were strained (40 μm, Corning) and plated on Geltrex coated plates at 150,000 cells/cm^2^ in the same media but without IL-3 for 14 days for macrophages or in Advanced DMEM/F12 (Invitrogen) with 100 ng/mL IL-34, 10 ng/mL GM-CSF, 1% N2 (Invitrogen), 2 mM Glutamax, penicillin/streptomycin and 0.055 mM β-mercaptoethanol for 14 days for microglia, with half media changes twice a week. Microglia were treated with 100 ng/ml LPS (L4391; Sigma-Aldrich) for 24 h.

### Differentiation of hiPSCs to motor neurons and astrocytes

Generation of hiPSC derived motor neurons and astrocytes was performed using a previously described protocol (Hall et al., 2017). Briefly, after neural conversion (7 days in a chemically defined medium containing 1 μM dorsomorphin (Millipore), 2 μM SB431542 (Tocris Bioscience), and 3.3 μM CHIR99021 (Miltenyi Biotec), neural precursors were patterned for 7 days with 0.5 μM retinoic acid and 1 μM purmorphamine, followed by a 4-day treatment with 0.1 μM purmorphamine. Motor neurons were terminally differentiated in 0.1 μM compound E for 3 days. For astrocytes, the patterned neural precursors above were subjected to a propagation phase (> 60 days) with 10 ng/mL FGF-2 (Gibco), after which the resulting glial precursors terminally differentiated to astrocytes in the presence of BMP4 (10 ng/mL, R&D) and LIF (10 ng/mL, Sigma-Aldrich) for 21 days followed by 7 days in N2B27 (DMEM/F12 Glutamax, Neurobasal, L-Glutamine, N2 supplement, non essential amino acids, B27 supplement, β-mercaptoethanol). Motor neurons and astrocytes were treated with microglia conditioned media at a ratio of 50:50 with fresh N2B27 (containing 0.1 μM compound E for motor neuron culture) for 72 h. For co-cultures, macrophage precursors were plated onto motor neurons at day 1 of terminal differentiation in 50:50 microglia and motor neuron media with 100 ng/mL IL-34, 10 ng/mL GM-CSF and 0.1 μM compound E. Co-cultures were aged to D10 before fixation.

### GPNMB siRNA treatment

Microglia were transfected using RNAiMAX (Thermo Fisher) to deliver 8.3 nM siRNAs targeting *GPNMB* (Horizon, ON-TARGETplus; L-011741–01) or a scrambled control siRNA (Horizon, ON-TARGETplus; D-001810–10) in Optimem overnight followed by a media change and then harvested or assayed 48 h later.

### RNA extraction and qPCR

The Promega Maxwell RSC simplyRNA cells kit including DNase treatment was used for RNA extractions using the Maxwell RSC instrument. A nanodrop was used to assess RNA concentration and the 260/280 ratio. RNA integrity was assessed using the Agilent TapeStation. All RNA samples had a RIN score of > 8.4. RT-qPCR was performed on cDNA generated from 200 ng DNaseI-treated total RNA using SuperScript® IV First-Strand Synthesis System (Invitrogen) and random hexamers, according to the manufacturer's instructions. RT-qPCR reactions were performed in 10 µl volumes containing 1 × SYBR® Green Mastermix (Bio-Rad) and 0.5 μM of the respective forward and reverse primers. *GPNMB* F’: TCCTGACAGAGACCCAGCC, R’: CACCAAGAGGGAGATCACAGT; *GAPDH* F’: ATGACATCAAGAAGGTGGTG, R’: CATACCAGGAAATGAGCTTG Samples were amplified and analysed using the CFX96™ Real Time PCRMachine (Bio-Rad). Cycling conditions were: 50 °C for 2 min, 95 °C for 2 min, followed by 40 cycles at 95 °C for 15 s, then 60 C for 60 s. Samples were run in duplicate and all programs contained a melt curve and a no template control. The absence of contaminating gDNA was confirmed by PCR on negative RT samples. Fold change was calculated using the delta *C*_T_ method.

### RNA Sequencing

Poly(A) + selected reverse stranded RNA sequencing libraries were prepared from 4 control and 3 VCP lines using the KAPA mRNA HyperPrep Library kit for Illumina®, with 50 ng of total RNA as input. Libraries were sequenced on the NovaSeq 6000 platform. Reads were processed using the nfcore/rna-seq v3.6 pipeline [94]. Raw reads underwent adaptor trimming with Trim Galore [95], removal of ribosomal RNA with SortMeRNA [96], alignment to Ensembl GRCh38.99 human reference genome using splice-aware aligner, STAR v2.7.1 [97] and BAM-level quantification with Salmon [98]. Detailed quality control of aligned reads was assessed utilising FastQC [99], RSeQC [100], Qualimap [101], dupRadar [102], Preseq [103] and MultiQC [104] tools. Differential gene expression was measured using DESeq2 [105] at the gene-level in R v4.2.0 [106]. We confirmed the identities of hiPSC microglia by clustering with RNA sequencing studies of cell subpopulations isolated and purified from the human brain [44]. Result contrasts were generated by comparing *VCP* mutant versus healthy control samples as well as LPS versus untreated samples using the Wald test. To mitigate the impact of parent cell line relatedness, we incorporated a covariate for the parent cell line in our statistical design, controlling for the inclusion of a control line edited to carry the *VCP*^*R191Q*^ mutation and an isogenic line with the *VCP*^*R155C*^ mutation corrected. Similarly, to account for cell line-specific effects in the comparison between LPS-stimulated and unstimulated samples, we included the cell line as a statistical term in the design, as each cell line had a paired LPS-stimulated and unstimulated sample.

Results for various analyses were correlated by matching the Wald test statistic for each gene followed by Pearson correlation. Genes were considered differentially expressed at FDR < 0.05. Significantly up- and down-regulated differentially expressed genes were used as input to functional over-representation analyses to identify enriched pathways using g:Profiler2. g:Profiler2 searches the following data sources: Gene Ontology (GO; molecular functions, biological processes and cellular components), KEGG, REAC, WikiPathways, CORUM and Human Phenotype Ontology. g:Profiler2 reports the hypergeometric test p-value with an adjustment for multiple testing using the Bonferroni correction. Over-represented function categories are plotted in bar charts, where the top significant terms were manually curated by removing redundant terms. GSEA was performed using the FGSEA package [107]. Overlap between 2 lists of genes was tested statistically using Fisher's exact test. The decoupleR package was used to estimate PROGENy signalling pathway activities and DoRothEA TF regulon activities inferred from gene expression changes [108].

### Proteomics

Microglial samples from 5 control and 5 *VCP* mutant lines, as well as 2 LPS-stimulated healthy control lines were reduced, alkylated and acetone precipitated overnight. Each protein pellet was resuspended in 1 M guanidine hydrochloride and 100 mM HEPES. Proteins were digested using trypsin overnight at 37 °C with mixing. Digested samples were acidified then stored at − 80 °C. Each sample was split into triplicates and loaded onto prepared Evotips. Samples were analysed using Evosep 15 cm column and an orbitrap Fusion mass spectrometer operating in data-dependent analysis (DDA) acquisition mode. A 44-min universal (OT/IT) method was used. Raw files were analysed with MaxQuant v1.6.12.0 using the LFQ algorithm against a 2020 SwissProt *Homo sapiens* protein database.

Mass spectrometry proteomics data were analysed using DEP (v1.11.0) [109] on MaxQuant results. Data was filtered, normalized and imputed using default parameters. Differential protein analysis was performed comparing *VCP* mutant versus control samples as well as LPS stimulated versus unstimulated samples using protein-wise linear models combined with Bayes statistics that utilises limma. A protein was considered significantly differentially expressed when FDR < 0.05. All error bars in the boxplots shown represent 1.5 times the interquartile range.

### Immunolabeling, imaging and analysis

Samples were fixed in 4% Paraformaldehyde and then blocked in 5% Bovine serum albumin (BSA) in PBS, 0.3% Triton X-100. Primary antibody for IBA1 (1:100; abcam, ab5076), P2RY12 (1:125; Atlas, HPA014518), TMEM119 (1:100; abcam, ab185333) or ISL1 (1:50; DSHB 40.2D6 Mouse IgG1) was added overnight at 4 °C in blocking solution. The next day wells were washed with PBS and incubated with secondary antibodies in blocking solution and 100 ng/mL 4′,6-diamidino-2-phenylindole DAPI nuclear stain for one hour. Images were acquired using the Opera Phenix High-Content Screening System (Perkin Elmer) with 40 × water objective as confocal z-stacks with z-step of 1um and were processed as maximum projection. For each well, 10 fields were acquired and analyzed using the Columbus Image Analysis System (Perkin Elmer).

#### Western blotting

Protein lysates were loaded onto NuPAGE, 4–12%, Bis–Tris protein gels (Invitrogen) and subjected to electrophoresis before being transferred onto nitrocellulose membrane (Biorad). Blocking was performed in PBS, 0.1% Tween 20, 5% bovine serum albumin for 1 h at room temperature and incubated overnight with either with rabbit anti-GPNMB (Cell Signalling; #E4D7P) or mouse anti-GAPDH (Cell Signalling; #D4C6R). Following washes, membranes were incubated for 1 h at room temperature fluorescent secondary anti-rabbit and anti-mouse antibodies (1:10,000, LI-COR IRDye). Membranes were imaged using Odyssey CLx Imaging System (LI-COR).

#### Cytokine and chemokine arrays

Media from hiPSC-derived microglial cultures was collected after 24 h and was analysed using the Procartaplex™ 25- PLEX, Human Cytokine assay (Thermo Fisher, LHC0009M) according to manufacturer's instructions. Samples were run in duplicate and analyzed on a Luminex 200 platform. Heat maps were produced using the log_2_ fold change of each cytokine or chemokine normalised to confluency.

For further analysis of CXCL10 and IL-6, media from hiPSC-derived microglia cultures was collected 72 h after transfection and analysed by CXCL10 (IP-10) or IL-6 ProQuantum immunoassay (Thermo Fisher, A35578 or A35573) according to manufacturer's instructions.

#### Live imaging

For labelling of acidic organelles and mitochondrial membrane potential, hiPSC-derived microglia were incubated with 1 μM Lysosensor Green DND-189 (Invitrogen), 75 nM Lysotracker Red (Invitrogen) or 20 nM TMRM (Tetramethylrhodamine, methyl ester) and a Hoechst stain in live cell imaging media (Gibco) for 30 min before imaging at 37 °C and 5% CO_2_ using the Opera Phenix High-Content Screening System (Perkin Elmer) with a 40 × water objective, as confocal z-stacks with z-step of 1um processed as maximum projections. Cathepsin D and cathepsin B activity were measured using 1 μM BODIPY FL-pepstatin A (invitrogen) and Magic red cathepsin B substrate (Biorad) according to manufacturer's instructions. Spot number, size and mean intensity measurements were taken as the average of 10 fields from each of triplicate wells using the Columbus Image Analysis System (Perkin Elmer). For measurement of phagocytosis, hiPSC-derived microglia were incubated with 0.1 mg/ml pHrodo green Bioparticles in live imaging media (Gibco) and immediately imaged once an hour for 6 h with the 10 × objective of a Incucyte (Sartorius) imaging platform. Total intensity measurements were averaged from 4 separate fields from triplicate wells. For measurement of neuronal death, hiPSC-derived motor neurons were incubated for 72 h with microglia conditioned media before media was changed into fresh N2B27 containing compound E and either 1:1000 caspase 3/7 green (Sartorius) or 1:200 NucView 488 caspase-3 dye (Sigma-Aldrich) and 1:1500 NucRed nuclear dye (Sartorius) and imaged using the 10 × objective of an Incucyte imaging platform 21 h after MG132 (100 nM; Bachem) was added to appropriate conditions.

## Supplementary Information

Supplementary Material 1: Figure S1: Gene expression characterisation of hiPSC microglia. (A) Allele depth ratio (mutant reads / reference reads + mutant reads) for each sample at VCP gene exon 5 loci for the R155C (red) and R191Q (blue) mutations. Cell line ctrl_R4 is the VCP R155C corrected line and vcp_R3 is the VCP R191Q inserted line. (B) Dendrogram of our hiPSC microglia (denoted Clarke) compared to publicly available hiPSC-derived microglia (Reich et al., 2021; Abud et al., 2017), primary human fetal and adult microglia (Abud et al., 2017) and other primary CNS cell types (Zhang et al., 2016). (C) Heatmap showing row-scaled normalised gene expression counts of the 249 genes from the core human microglia transcriptional signature [45] across the same samples. (D) Quantification of morphology by linear classifier of round, bipolar, multipolar or secondary branched morphology based on [38]. Error bars represent standard error of mean. Data points are individual cell lines (3-5 lines per condition from 2 experimental blocks, each point indicates an average of 2 technical repeats).

Supplementary Material 2: Figure S2: VCP mutant microglia transcriptomic and functional signatures. (A) Scatterplot of VCP mutant versus CTRL gene expression changes (test statistic) in all samples (X-axis) against non-isogenic microglia (y-axis). Overlapping differentially genes are coloured red. The solid blue line represents the linear correlation and Pearson correlation *R* = +0.7. (B) Venn overlap of differentially expressed genes in all samples and non-isogenic samples. (C) PROGENy signaling pathway activity normalized enrichment scores (y -axis) in VCP mutant microglia. (D) DoRothEA transcription factor regulon analysis in VCP mutant microglia. Gene set enrichment analysis for (E) phagocytosis genes in VCP mutant microglia. (F) Representative images and (G) total integrated intensity image quantification of phagocytosis of pHrodo conjugated bioparticles for healthy control and VCP mutant microglia. Data points are the average of the mean of 1-3 technical repeats from 3 experimental blocks, 2-3 lines per condition from 2 differentiations. Gene set enrichment analysis for (H) mitochondrial genes in VCP mutant microglia. (I) Representative images, (J) mean TMRM intensity measurements and (K) mitochondrial mass measurements in CTRL and VCP mutant microglia. Scale bar: 50μm. Data points are individual cell lines (3-4 lines per condition from 1 differentiation, average of 2-3 technical repeats) from 3 experimental blocks. Data are 3-4 lines per condition from 1 differentiation including 2 isogenic pairs (1 inserted R191Q mutation and 1 isogenic corrected R155C mutation).

Supplementary Material 3: Figure S3: Lysosomal phenotypes are not observed in VCP mutant microglia precursors or hiPSCs. Quantification of control and VCP mutant hiPSCs and microglia precursors (A) Lysosensor intensity and Lysotracker intensity (B), spot area (C) and spot number per area of cytoplasm (D). *N* = 3-6, 3 experimental blocks.

Supplementary Material 4: Figure S4: LPS stimulation induces reactive changes in hiPSC microglia. (A)Principal component analysis (PCA) of variance stabilised counts plotted by their coordinates along the first two principal components for untreated and LPS-stimulated VCP mutant and healthy control microglia. (B) Volcano plot of log_2_ fold change in differential gene expression between untreated and LPS-stimulated healthy control microglia. (C)GO terms enriched in upregulated (red) and downregulated (blue) differentially expressed genes in LPS-stimulated microglia. (D) PROGENy signaling pathway activity normalized enrichment scores (y -axis) in LPS-stimulated microglia. (E) DoRothEA transcription factor regulon analysis in LPS-stimulated microglia. Data are from 4 healthy control lines per condition from 1 differentiation. (F) Volcano plot of log_2_ fold change in differential protein expression between untreated and LPS-stimulated healthy control microglia. (G) GO terms enriched in upregulated (red) and downregulated (blue) differentially expressed proteins in LPS-stimulated microglia. (H) Heatmap showing differentially secreted cytokines and chemokines in LPS-stimulated microglia. Data are from 5 healthy control lines from 1 differentiation.

Supplementary Material 5: Figure S5: GPNMB knockdown reduces inflammatory signalling and phagocytosis in hiPSC microglia. (A) qPCR of GPNMB expression in GPNMB or scrambled siRNA treated healthy control and VCP mutant microglia. (B) RNAseq showing Transcripts Per Million (TPM) of GPNMB expression in GPNMB or scrambled siRNA treated healthy control and VCP mutant microglia. (C) GPNMB expression measured by qPCR in hiPSC, microglia precursors, and microglia. Data are from 3-6 lines per condition from 3 experimental blocks. Quantification of GPNMB cytoplasmic (D) and membrane intensity (E), GPNMB puncta size (F) and number (G) and representative images (H). Scale bar 50μm. Data are from 3-5 lines per condition from 3 experimental blocks from 1 differentiation. (I) Volcano plot of log_2_ fold change in differential gene expression between GPNMB and scrambled siRNA treated healthy control microglia. (J) PROGENy signaling pathway activity normalized enrichment scores (y -axis) in GPNMB siRNA treated healthy control microglia. (K) DoRothEA transcription factor regulon analysis in GPNMB siRNA treated healthy control microglia. Data are from 3 lines per condition from 1 differentiation. (L) Representative images and (M) quantification for pHrodo conjugated bioparticles (green) in hiPSC microglia treated with scrambled or GPNMB siRNA. Scale bar 200μm. Statistics are from a generalised linear model comparing the 3 treatment groups at each timepoint accounting for cell line and experimental repeat; * *p* < 0.05.

Supplementary Material 6: Figure S6. GPNMB knockdown does not affect microglial lysosomal function. A Representative images of Lysosensor (cyan) and Hoechst (blue) in control and VCP mutant microglia treated with scrambled or GPNMB siRNA. Scale bar: 50 um. (B) Quantification of Lysosensor mean intensity. (C) Representative images of Lysotracker (magenta) and Hoechst (blue) in control and VCP mutant microglia treated with scrambled or GPNMB siRNA. Scale bar: 50 um. Quantification of Lysotracker mean intensity (D) spot area (E) and number of spots per area (F). (G) Representative images of pepstatin A (cyan) and Hoechst (blue) in control and VCP mutant microglia treated with scrambled or GPNMB siRNA. Scale bar: 50 um. (H) Quantification of pepstatin A (cathepsin D activity) mean intensity. (I) Representative images of Magic Red (magenta) and Hoechst (blue) in control and VCP mutant microglia treated with scrambled or GPNMB siRNA. Scale bar: 50 um. (J) Quantification of Magic Red (cathepsin B activity) mean intensity. *N *=3-5, 3 experimental blocks from 1 differentiation.

Supplementary Material 7: Figure S7: Non cell autonomous effects of VCP mutant and LPS-stimulated microglia on hiPSC derived healthy control motor neurons and astrocytes. (A) Representative images for caspase 3/7 (green) and NucRed (red) staining in healthy control motor neurons treated with healthy control or VCP mutant microglia conditioned media. Scale bar: 200μm. (B) Quantification of motor neuron death in healthy control or VCP mutant microglia conditioned media treated motor neurons expressed as the change in death from motor neurons in fresh media per line. (C) PROGENy signaling pathway activity normalized enrichment scores (y -axis) in LPS-stimulated motor neurons. (D) DoRothEA transcription factor regulon analysis in LPS-stimulated motor neurons. (E) PROGENy signaling pathway activity normalized enrichment scores (y -axis) in LPS-stimulated astrocytes. (F) DoRothEA transcription factor regulon analysis in LPS-stimulated astrocytes. Volcano plot of log_2_ fold change in differential gene expression between LPS-stimulated and unstimulated microglia conditioned media treated (G) motor neurons and (H) astrocytes. (I) PROGENy signaling pathway activity normalized enrichment scores (y -axis) in LPS-stimulated microglia conditioned media treated motor neurons. (J) DoRothEA transcription factor regulon analysis in LPS-stimulated microglia conditioned media treated motor neurons. (K) PROGENy signaling pathway activity normalized enrichment scores (y -axis) in LPS-stimulated microglia conditioned media treated astrocytes. (L) DoRothEA transcription factor regulon analysis in LPS-stimulated microglia conditioned media treated astrocytes. Data are from 3 healthy control lines per condition from 1 differentiation for motor neurons and 4 healthy control lines per condition from 1 differentiation for astrocytes.

Supplementary Material 8: Figure S8: Microglial GPNMB knockdown has no effect on motor neuron survival. Quantification of control and VCP mutant motor neuron survival under basal conditions (A) or after treatment with MG132 (B) for 21 hours after pretreatment with conditioned media from healthy control or VCP mutant microglia treated with scrambled control or GPNMB targeting siRNA. Scale bar: 200μm. Data are from 4 lines from 2-4 differentiations. Stats from generalised linear model accounting for cell line and experimental repeat.

Supplementary Material 9: Figure S9. Microglial GPNMB knockdown has no effect on motor neuron survival. (A) Representative images of immunofluorescence for cleaved caspase-3 (magenta), ISL1 (grey), IBA1 (cyan) and staining for DAPI (blue) from control and VCP mutant microglia and motor neuron co-cultures treated with scrambled or GPNMB siRNA. Scale bar: 50 um. (B) Quantification of proportions of ISL1 positive motor neurons and IBA1 positive microglia in control and VCP mutant microglia and motor neuron co-cultures treated with scrambled or GPNMB siRNA. Quantification of cleaved caspase-3 positive (C) and pyknotic motor neurons in control and VCP mutant microglia and motor neuron co-cultures treated with scrambled or GPNMB siRNA. Data are *N* =3 motor neuron and *N* =3 microglia lines per genotype from 1 differentiation.

Supplementary Material 10: Tables S1-19.

Supplementary Material 11: Tables S20-29.

## Data Availability

Raw sequencing data used in this study are available at GEO with accession number GSE282665. The mass spectrometry proteomics data have been deposited in the ProteomeXchange Consortium via the PRIDE partner repository with the dataset identifier PXD057586. Full code to reproduce analyses and figures is available through GitHub at https://github.com/ojziff/vcp_microglia.
