# Supplementary figures and images for: Human VCP mutant ALS/FTD microglia display immune and lysosomal phenotypes independently of GPNMB
Source: Mol Neurodegener. 2024 Nov 26;19:90. doi: 10.1186/s13024-024-00773-1 (PMC11590569; doi:10.1186/s13024-024-00773-1)

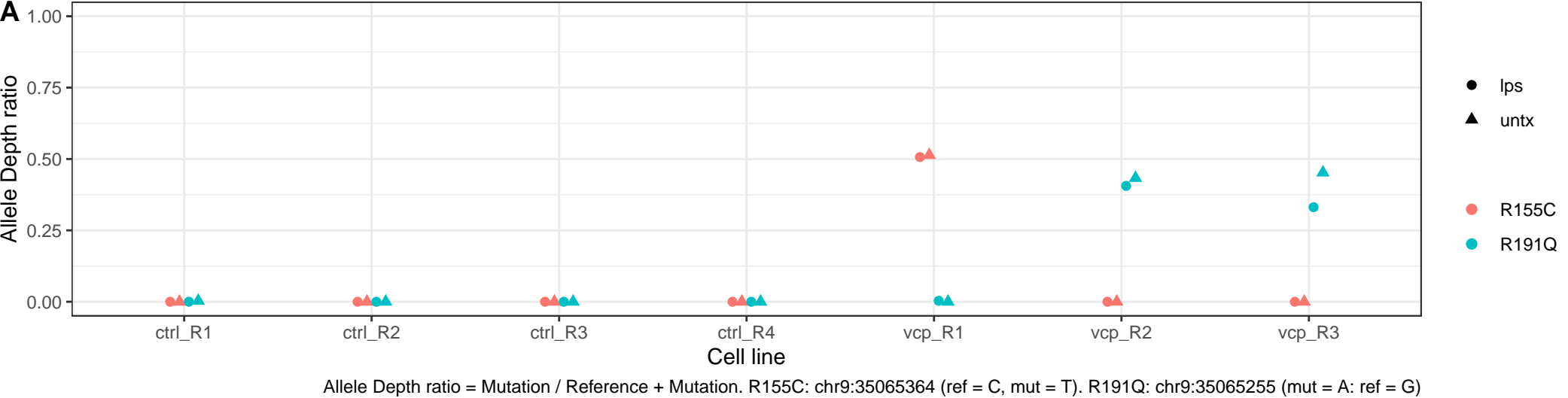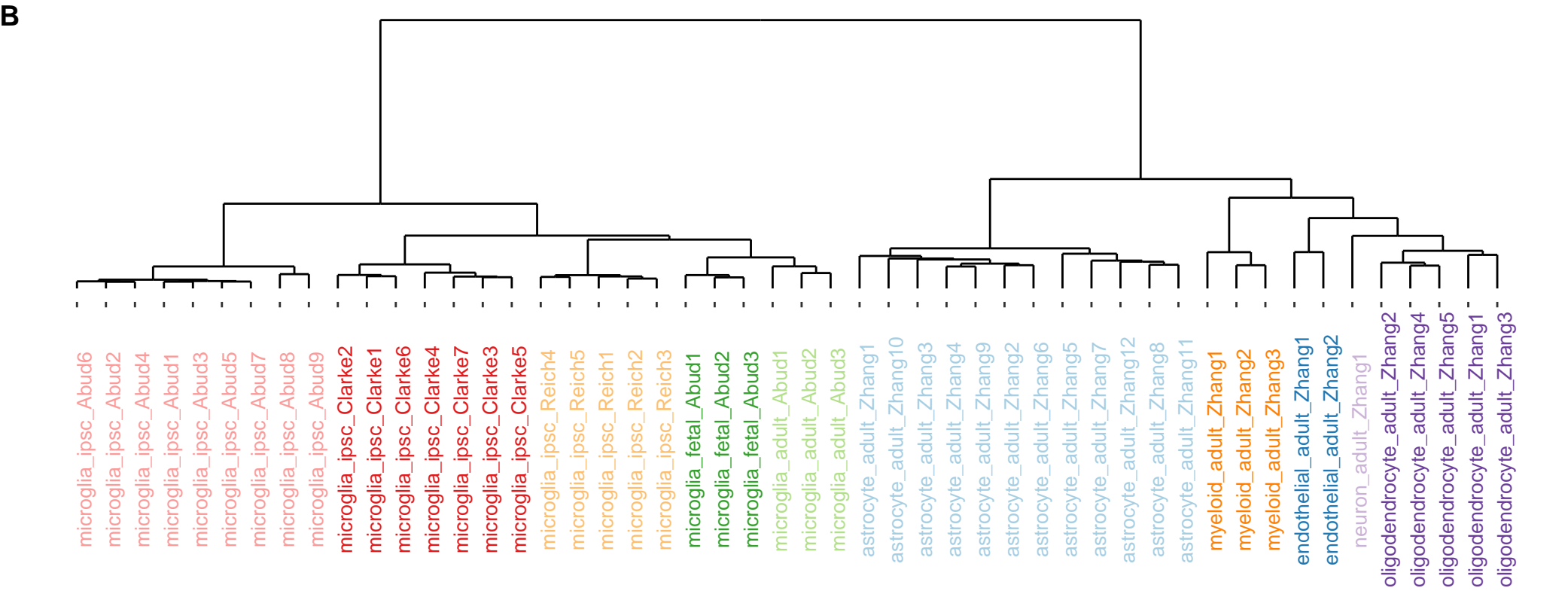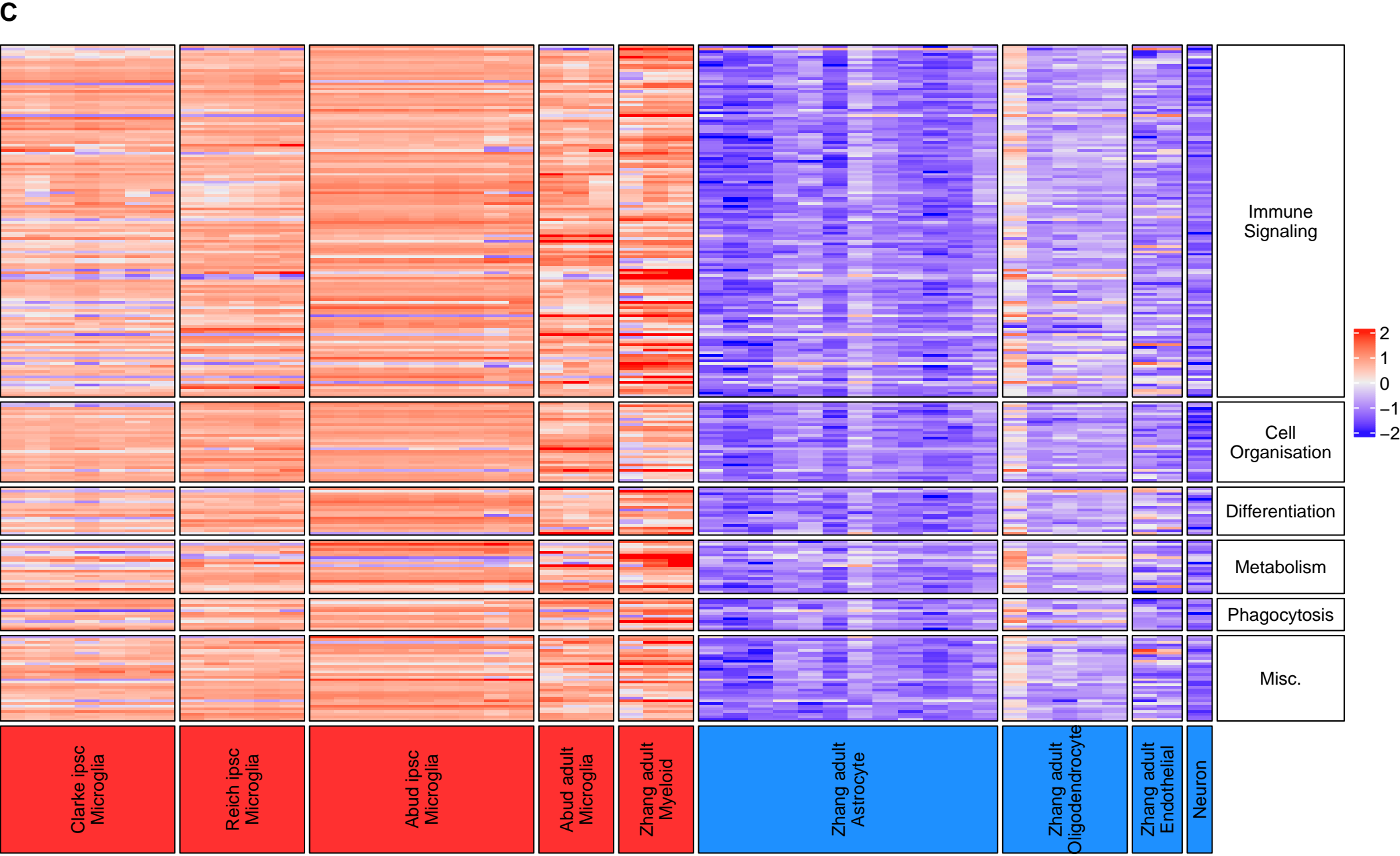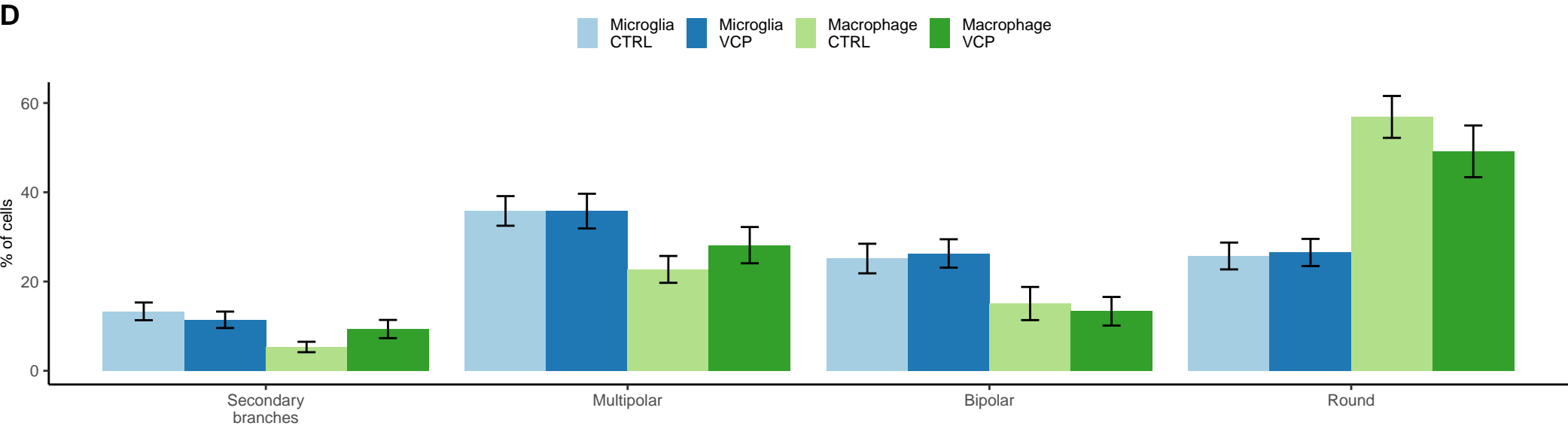

Supplement: Supplementary file 1 — Supplementary Material 1: Figure S1: Gene expression characterisation of hiPSC microglia. (A) Allele depth ratio (mutant reads / reference reads + mutant reads) for each sample at VCP gene exon 5 loci for the R155C (red) and R191Q (blue) mutations. Cell line ctrl_R4 is the VCP R155C corrected line and vcp_R3 is the VCP R191Q inserted line. (B) Dendrogram of our hiPSC microglia (denoted Clarke) compared to publicly available hiPSC-derived microglia (Reich et al., 2021; Abud et al., 2017), primary human fetal and adult microglia (Abud et al., 2017) and other primary CNS cell types (Zhang et al., 2016). (C) Heatmap showing row-scaled normalised gene expression counts of the 249 genes from the core human microglia transcriptional signature [45] across the same samples. (D) Quantification of morphology by linear classifier of round, bipolar, multipolar or secondary branched morphology based on [38]. Error bars represent standard error of mean. Data points are individual cell lines (3-5 lines per condition from 2 experimental blocks, each point indicates an average of 2 technical repeats). [file 13024_2024_773_MOESM1_ESM.pdf]

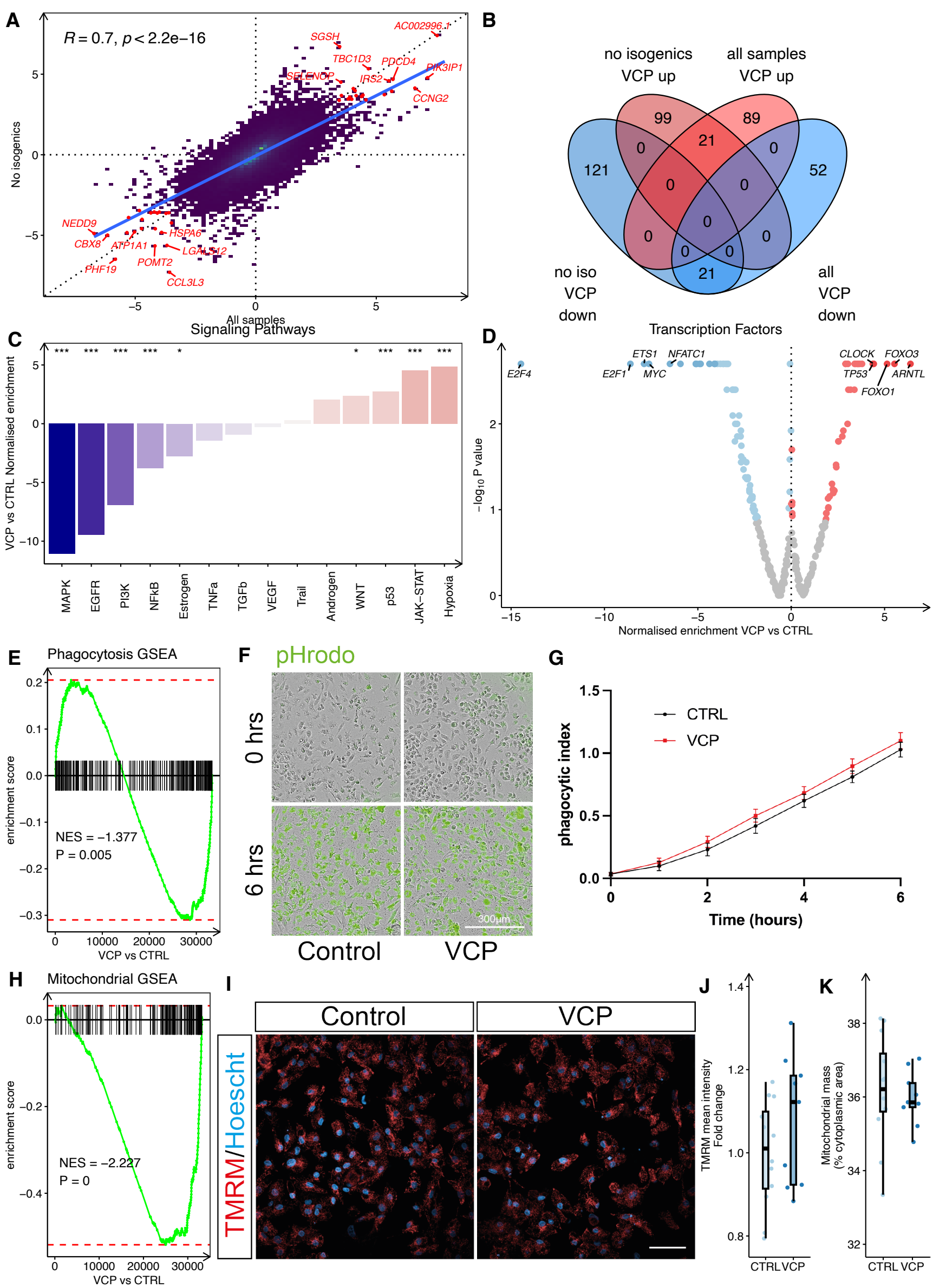

Supplement: Supplementary file 2 — Supplementary Material 2: Figure S2: VCP mutant microglia transcriptomic and functional signatures. (A) Scatterplot of VCP mutant versus CTRL gene expression changes (test statistic) in all samples (X-axis) against non-isogenic microglia (y-axis). Overlapping differentially genes are coloured red. The solid blue line represents the linear correlation and Pearson correlation R = +0.7. (B) Venn overlap of differentially expressed genes in all samples and non-isogenic samples. (C) PROGENy signaling pathway activity normalized enrichment scores (y -axis) in VCP mutant microglia. (D) DoRothEA transcription factor regulon analysis in VCP mutant microglia. Gene set enrichment analysis for (E) phagocytosis genes in VCP mutant microglia. (F) Representative images and (G) total integrated intensity image quantification of phagocytosis of pHrodo conjugated bioparticles for healthy control and VCP mutant microglia. Data points are the average of the mean of 1-3 technical repeats from 3 experimental blocks, 2-3 lines per condition from 2 differentiations. Gene set enrichment analysis for (H) mitochondrial genes in VCP mutant microglia. (I) Representative images, (J) mean TMRM intensity measurements and (K) mitochondrial mass measurements in CTRL and VCP mutant microglia. Scale bar: 50μm. Data points are individual cell lines (3-4 lines per condition from 1 differentiation, average of 2-3 technical repeats) from 3 experimental blocks. Data are 3-4 lines per condition from 1 differentiation including 2 isogenic pairs (1 inserted R191Q mutation and 1 isogenic corrected R155C mutation). [file 13024_2024_773_MOESM2_ESM.pdf]

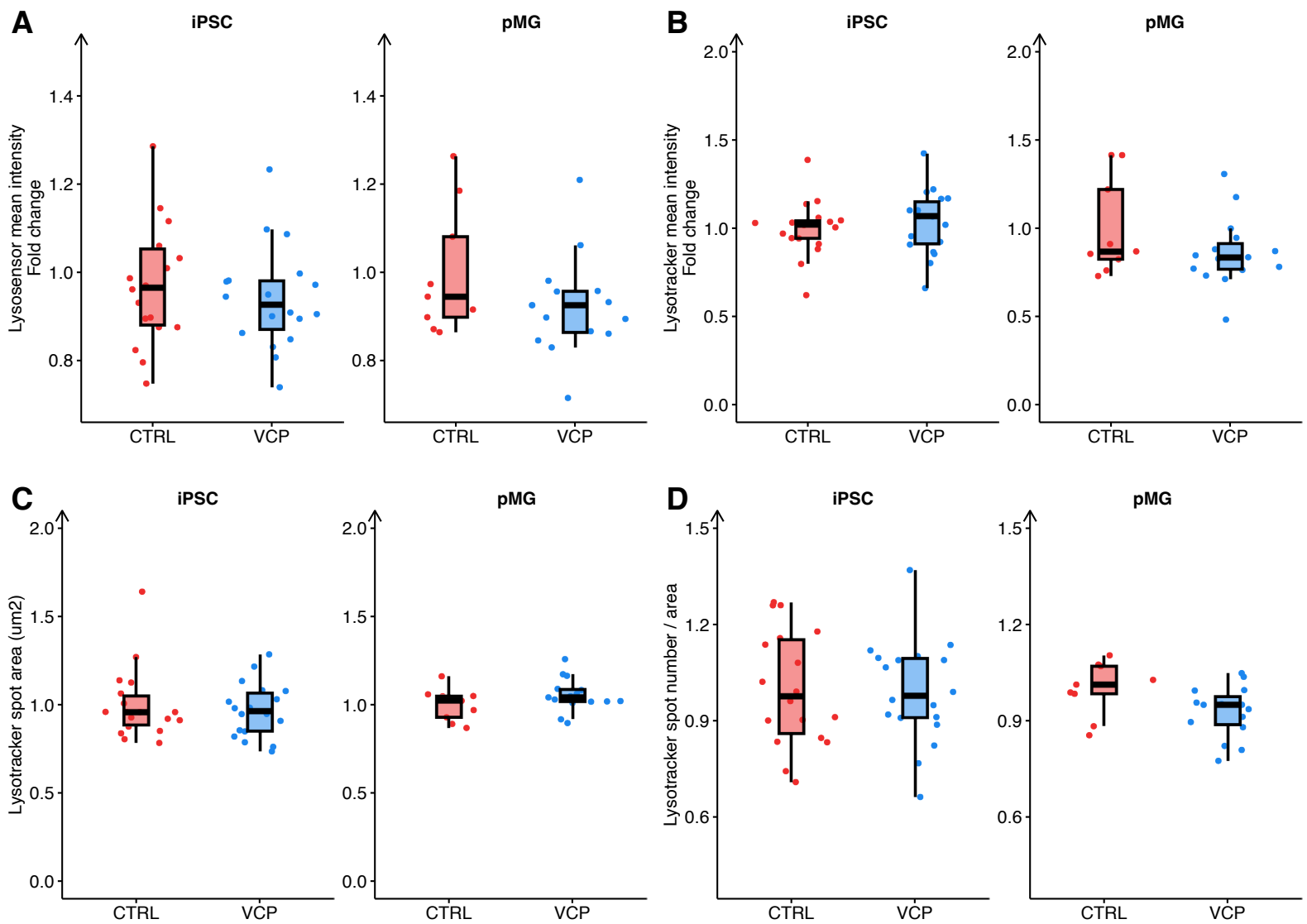

Supplement: Supplementary file 3 — Supplementary Material 3: Figure S3: Lysosomal phenotypes are not observed in VCP mutant microglia precursors or hiPSCs. Quantification of control and VCP mutant hiPSCs and microglia precursors (A) Lysosensor intensity and Lysotracker intensity (B), spot area (C) and spot number per area of cytoplasm (D). N = 3-6, 3 experimental blocks. [file 13024_2024_773_MOESM3_ESM.pdf]

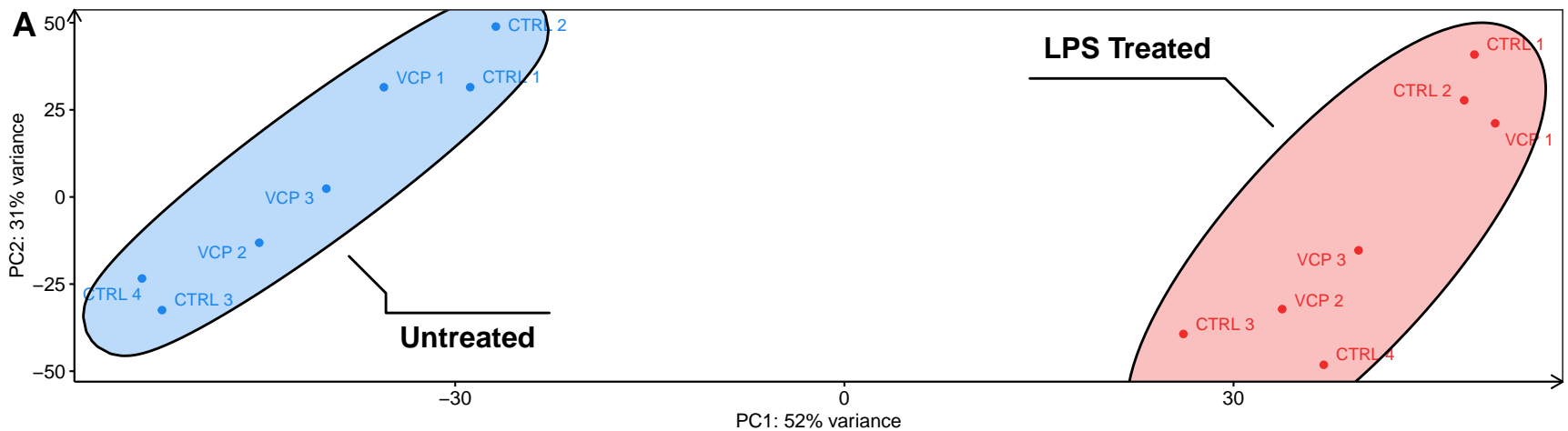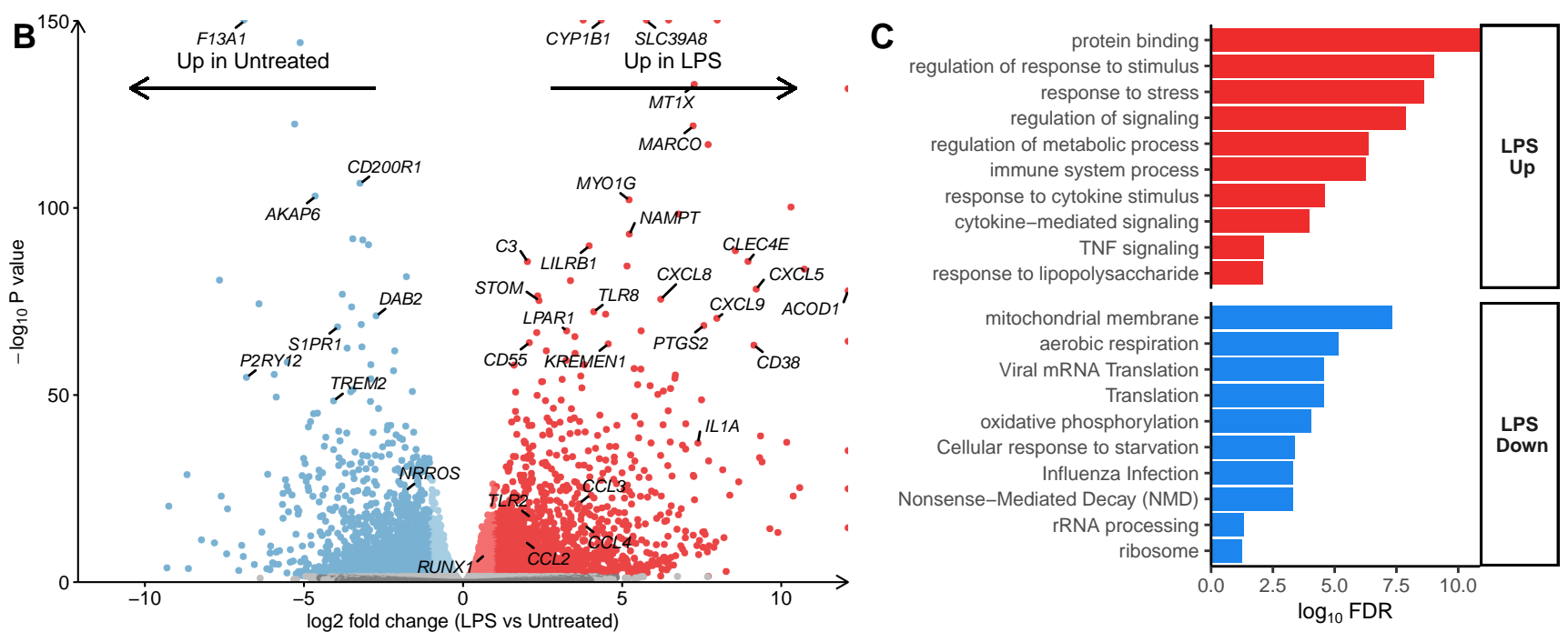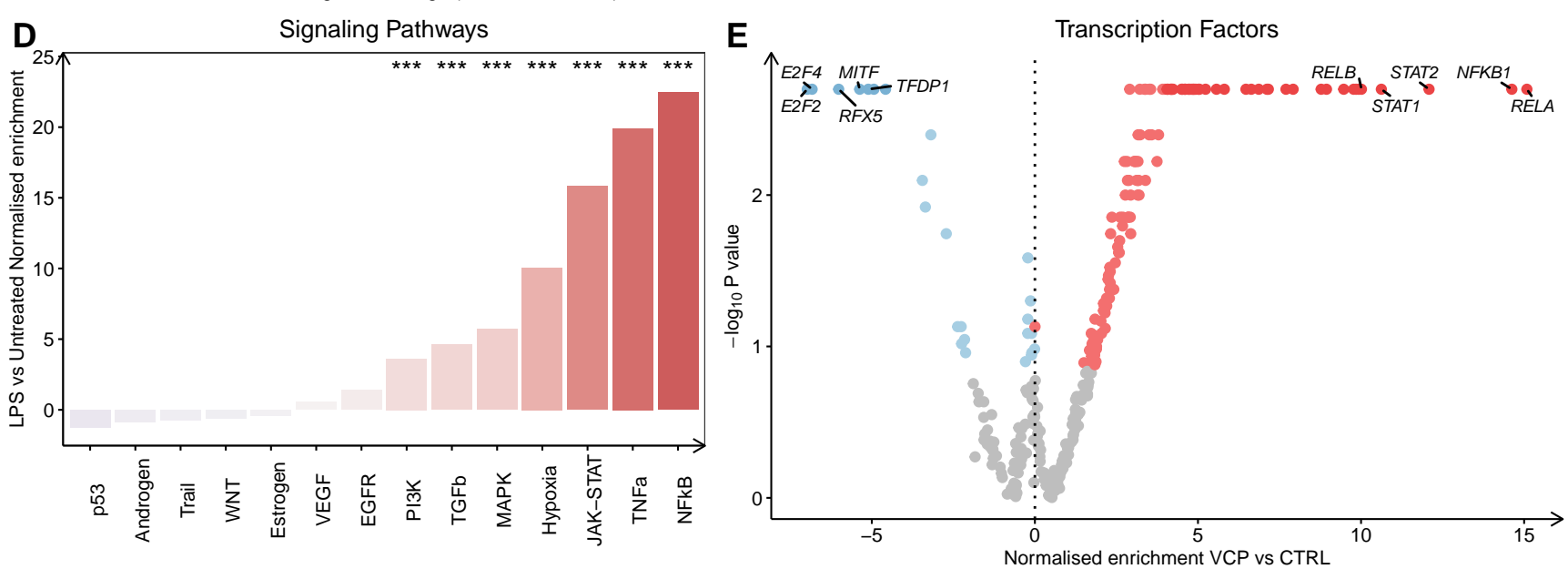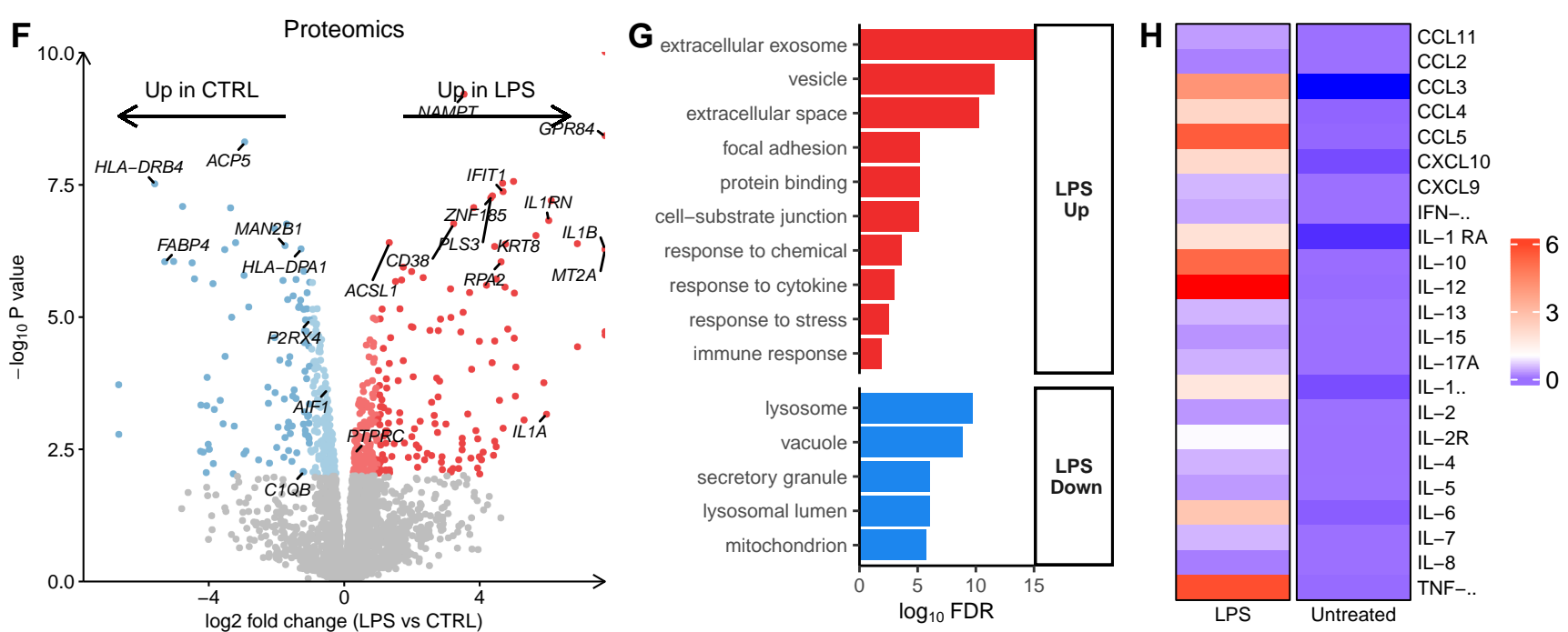

Supplement: Supplementary file 4 — Supplementary Material 4: Figure S4: LPS stimulation induces reactive changes in hiPSC microglia. (A)Principal component analysis (PCA) of variance stabilised counts plotted by their coordinates along the first two principal components for untreated and LPS-stimulated VCP mutant and healthy control microglia. (B) Volcano plot of log2 fold change in differential gene expression between untreated and LPS-stimulated healthy control microglia. (C)GO terms enriched in upregulated (red) and downregulated (blue) differentially expressed genes in LPS-stimulated microglia. (D) PROGENy signaling pathway activity normalized enrichment scores (y -axis) in LPS-stimulated microglia. (E) DoRothEA transcription factor regulon analysis in LPS-stimulated microglia. Data are from 4 healthy control lines per condition from 1 differentiation. (F) Volcano plot of log2 fold change in differential protein expression between untreated and LPS-stimulated healthy control microglia. (G) GO terms enriched in upregulated (red) and downregulated (blue) differentially expressed proteins in LPS-stimulated microglia. (H) Heatmap showing differentially secreted cytokines and chemokines in LPS-stimulated microglia. Data are from 5 healthy control lines from 1 differentiation. [file 13024_2024_773_MOESM4_ESM.pdf]

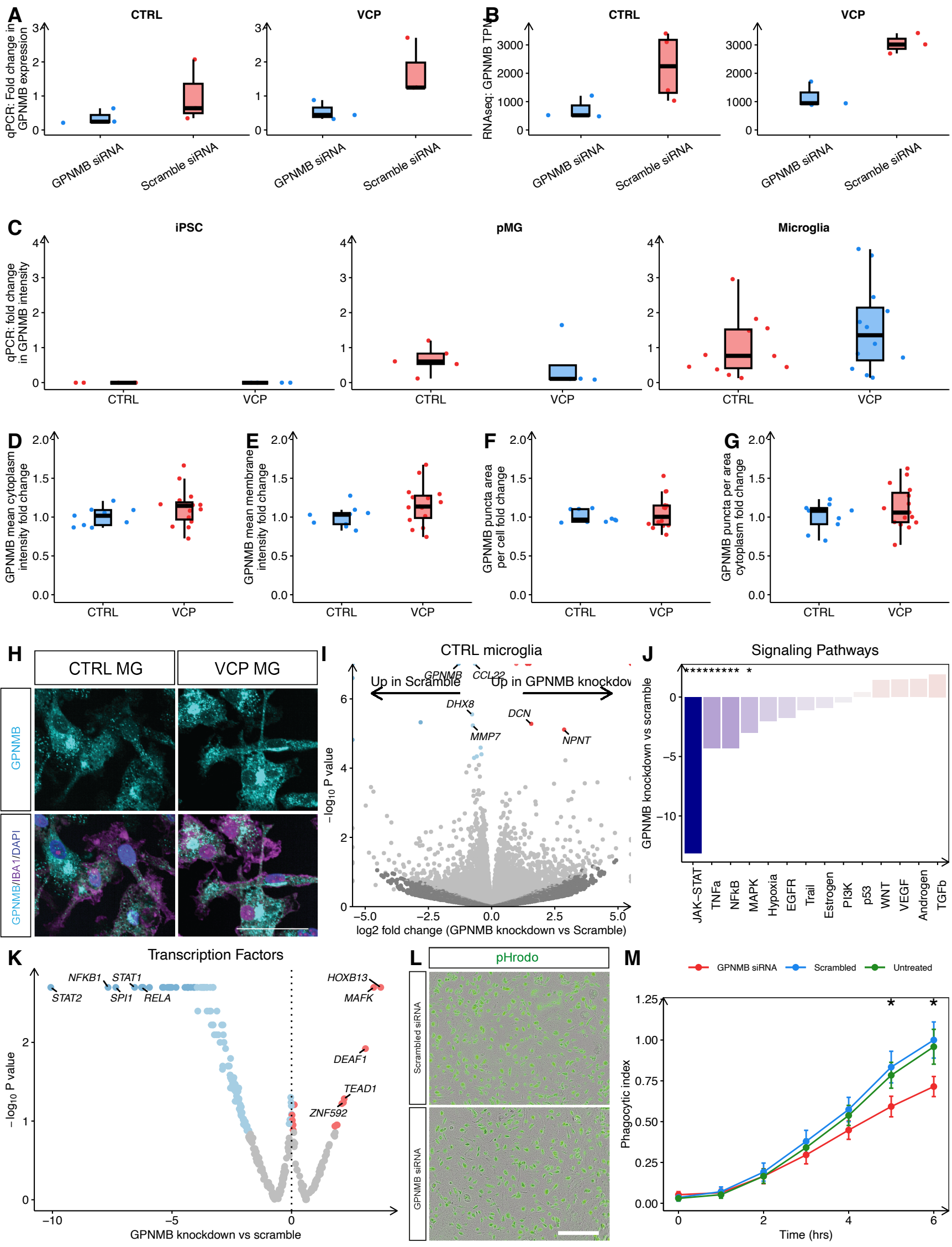

Supplement: Supplementary file 5 — Supplementary Material 5: Figure S5: GPNMB knockdown reduces inflammatory signalling and phagocytosis in hiPSC microglia. (A) qPCR of GPNMB expression in GPNMB or scrambled siRNA treated healthy control and VCP mutant microglia. (B) RNAseq showing Transcripts Per Million (TPM) of GPNMB expression in GPNMB or scrambled siRNA treated healthy control and VCP mutant microglia. (C) GPNMB expression measured by qPCR in hiPSC, microglia precursors, and microglia. Data are from 3-6 lines per condition from 3 experimental blocks. Quantification of GPNMB cytoplasmic (D) and membrane intensity (E), GPNMB puncta size (F) and number (G) and representative images (H). Scale bar 50μm. Data are from 3-5 lines per condition from 3 experimental blocks from 1 differentiation. (I) Volcano plot of log2 fold change in differential gene expression between GPNMB and scrambled siRNA treated healthy control microglia. (J) PROGENy signaling pathway activity normalized enrichment scores (y -axis) in GPNMB siRNA treated healthy control microglia. (K) DoRothEA transcription factor regulon analysis in GPNMB siRNA treated healthy control microglia. Data are from 3 lines per condition from 1 differentiation. (L) Representative images and (M) quantification for pHrodo conjugated bioparticles (green) in hiPSC microglia treated with scrambled or GPNMB siRNA. Scale bar 200μm. Statistics are from a generalised linear model comparing the 3 treatment groups at each timepoint accounting for cell line and experimental repeat; * p < 0.05. [file 13024_2024_773_MOESM5_ESM.pdf]

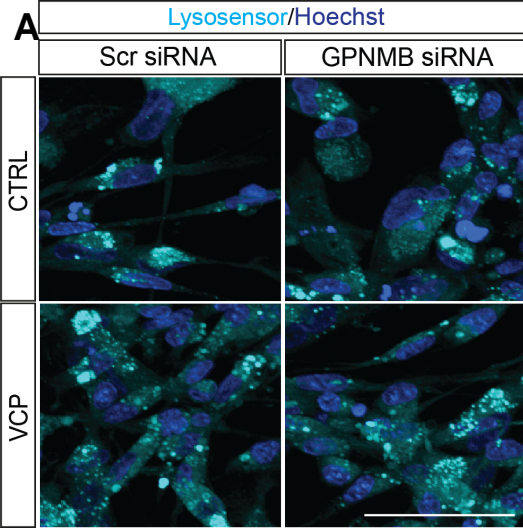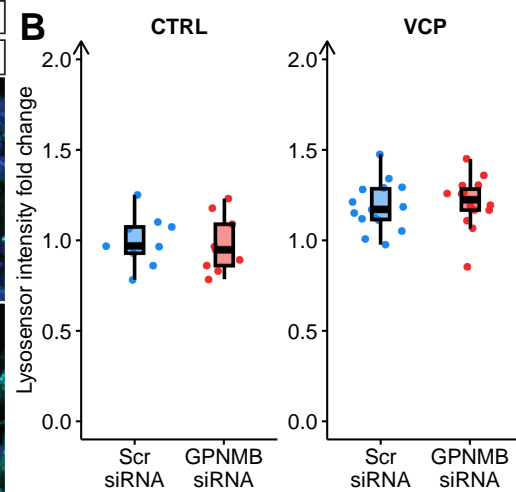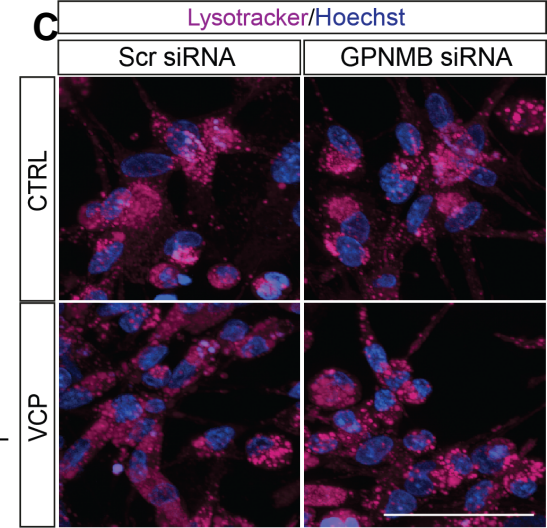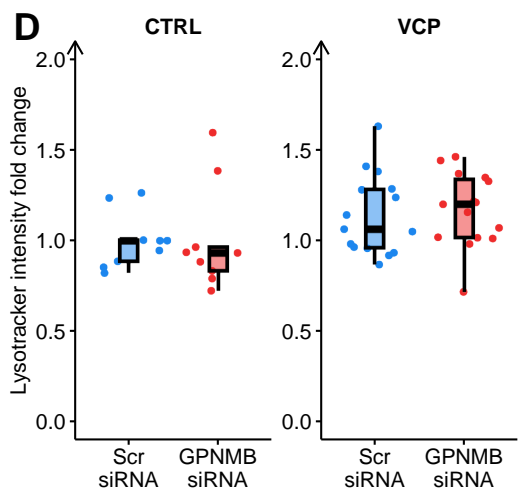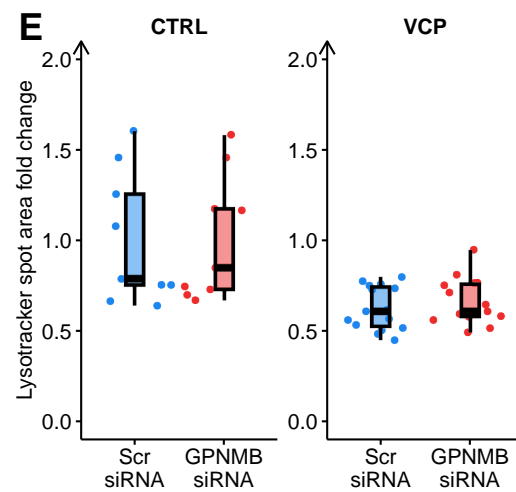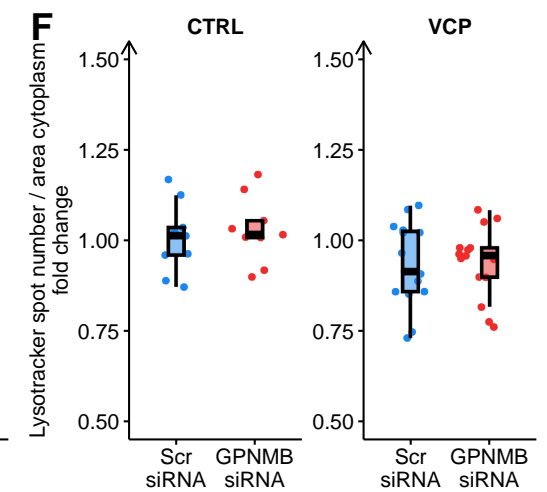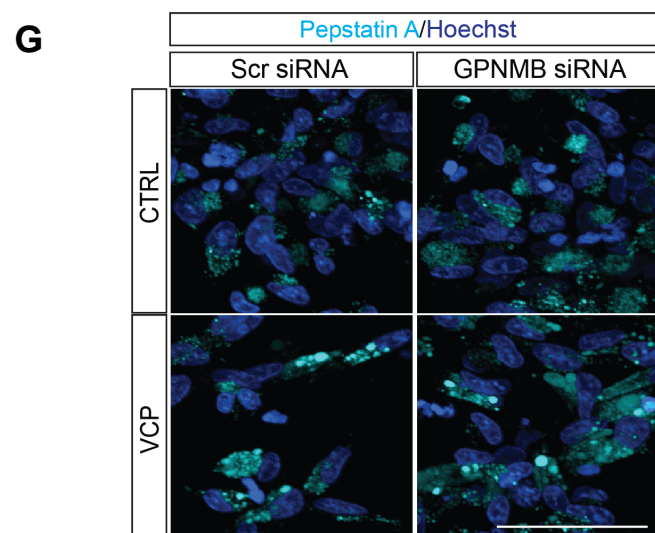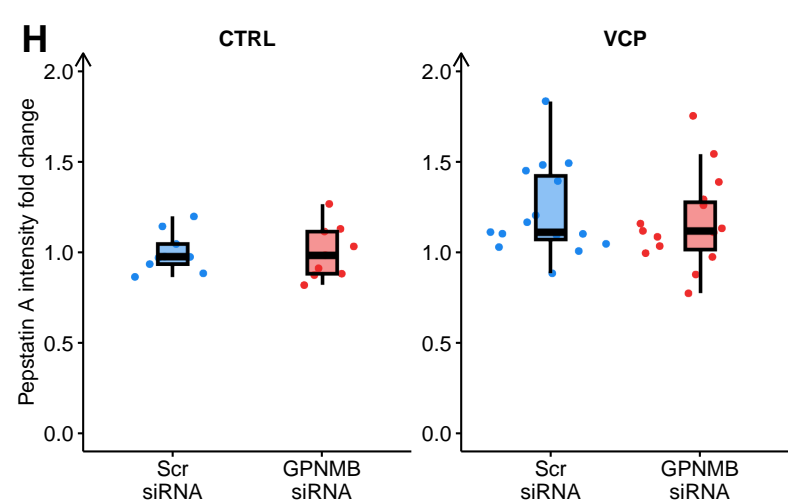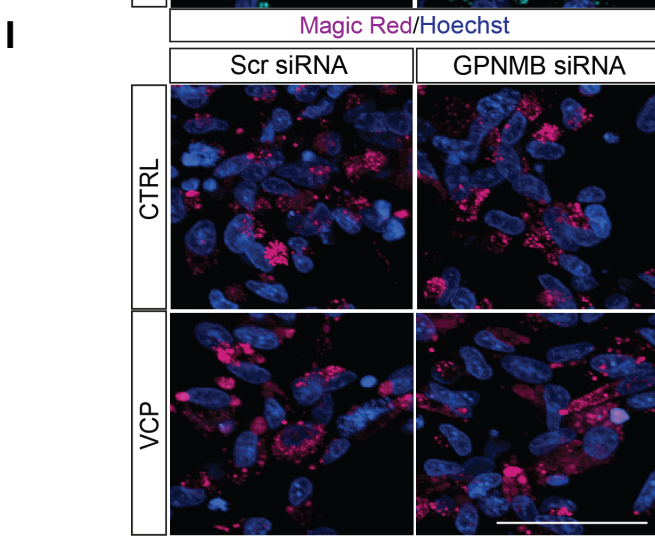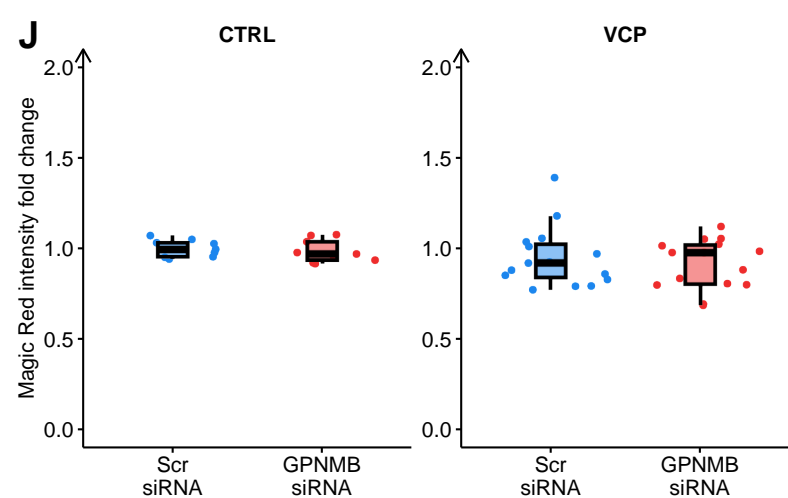

Supplement: Supplementary file 6 — Supplementary Material 6: Figure S6. GPNMB knockdown does not affect microglial lysosomal function. A Representative images of Lysosensor (cyan) and Hoechst (blue) in control and VCP mutant microglia treated with scrambled or GPNMB siRNA. Scale bar: 50 um. (B) Quantification of Lysosensor mean intensity. (C) Representative images of Lysotracker (magenta) and Hoechst (blue) in control and VCP mutant microglia treated with scrambled or GPNMB siRNA. Scale bar: 50 um. Quantification of Lysotracker mean intensity (D) spot area (E) and number of spots per area (F). (G) Representative images of pepstatin A (cyan) and Hoechst (blue) in control and VCP mutant microglia treated with scrambled or GPNMB siRNA. Scale bar: 50 um. (H) Quantification of pepstatin A (cathepsin D activity) mean intensity. (I) Representative images of Magic Red (magenta) and Hoechst (blue) in control and VCP mutant microglia treated with scrambled or GPNMB siRNA. Scale bar: 50 um. (J) Quantification of Magic Red (cathepsin B activity) mean intensity. N =3-5, 3 experimental blocks from 1 differentiation. [file 13024_2024_773_MOESM6_ESM.pdf]

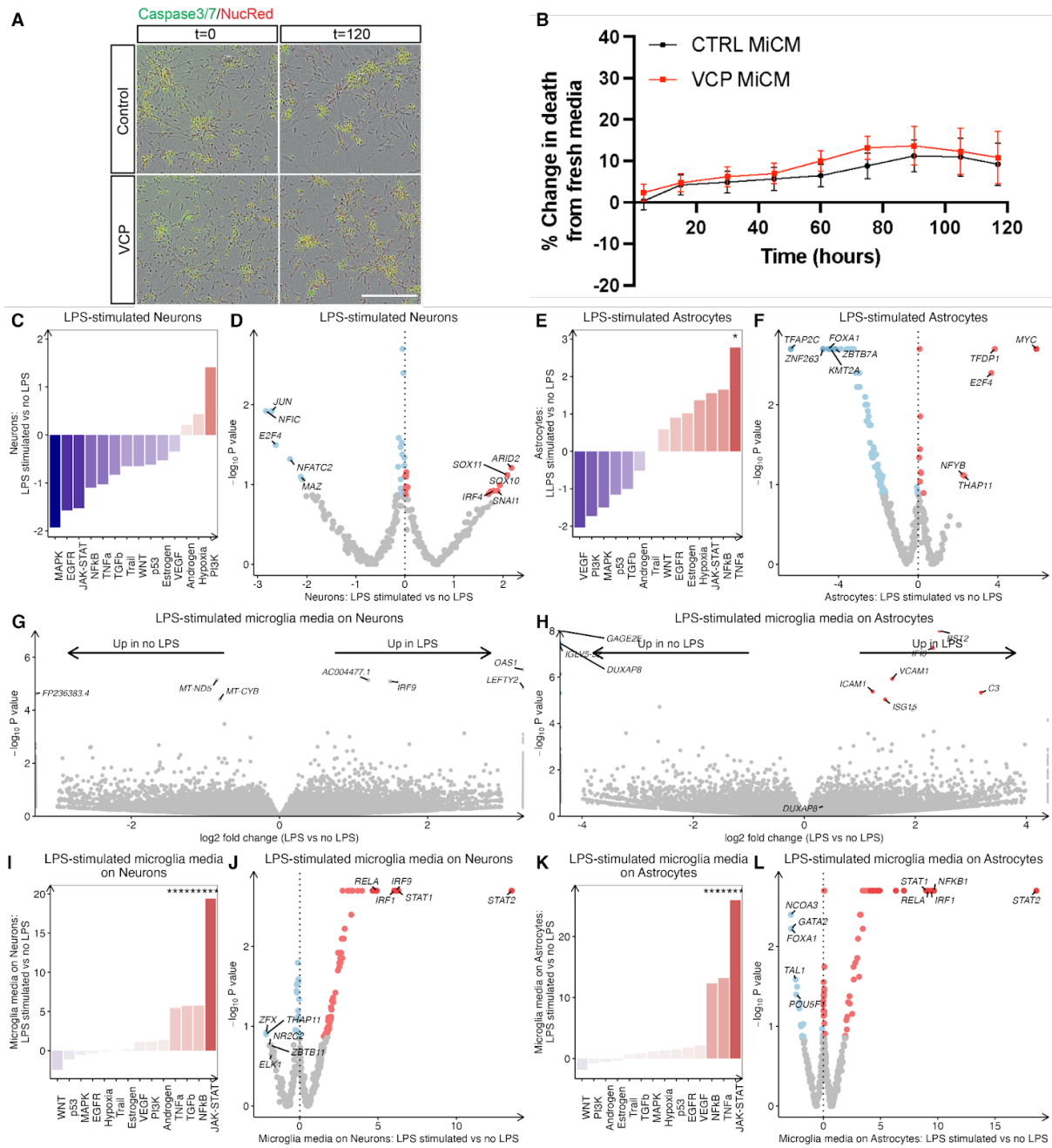

Supplement: Supplementary file 7 — Supplementary Material 7: Figure S7: Non cell autonomous effects of VCP mutant and LPS-stimulated microglia on hiPSC derived healthy control motor neurons and astrocytes. (A) Representative images for caspase 3/7 (green) and NucRed (red) staining in healthy control motor neurons treated with healthy control or VCP mutant microglia conditioned media. Scale bar: 200μm. (B) Quantification of motor neuron death in healthy control or VCP mutant microglia conditioned media treated motor neurons expressed as the change in death from motor neurons in fresh media per line. (C) PROGENy signaling pathway activity normalized enrichment scores (y -axis) in LPS-stimulated motor neurons. (D) DoRothEA transcription factor regulon analysis in LPS-stimulated motor neurons. (E) PROGENy signaling pathway activity normalized enrichment scores (y -axis) in LPS-stimulated astrocytes. (F) DoRothEA transcription factor regulon analysis in LPS-stimulated astrocytes. Volcano plot of log2 fold change in differential gene expression between LPS-stimulated and unstimulated microglia conditioned media treated (G) motor neurons and (H) astrocytes. (I) PROGENy signaling pathway activity normalized enrichment scores (y -axis) in LPS-stimulated microglia conditioned media treated motor neurons. (J) DoRothEA transcription factor regulon analysis in LPS-stimulated microglia conditioned media treated motor neurons. (K) PROGENy signaling pathway activity normalized enrichment scores (y -axis) in LPS-stimulated microglia conditioned media treated astrocytes. (L) DoRothEA transcription factor regulon analysis in LPS-stimulated microglia conditioned media treated astrocytes. Data are from 3 healthy control lines per condition from 1 differentiation for motor neurons and 4 healthy control lines per condition from 1 differentiation for astrocytes. [file 13024_2024_773_MOESM7_ESM.pdf]

**A**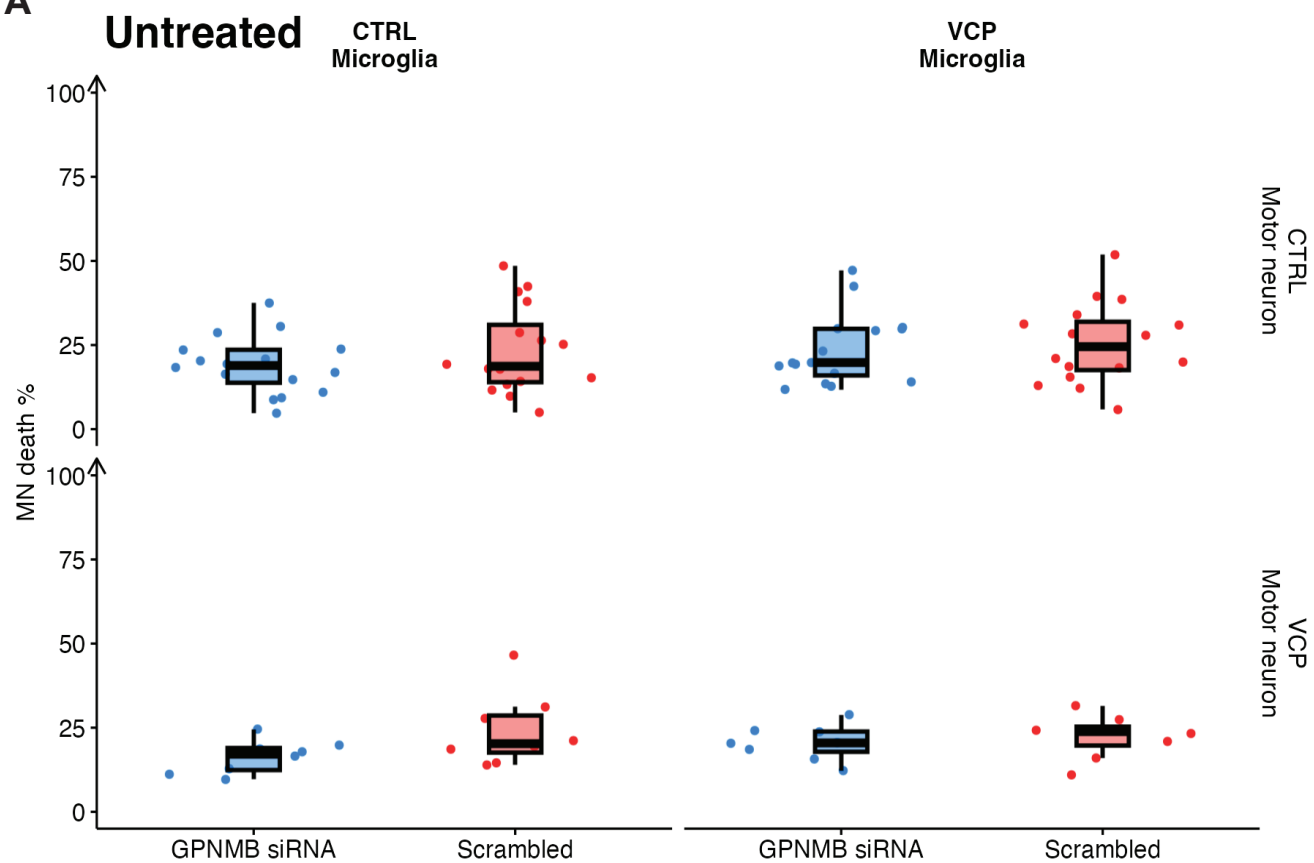**B**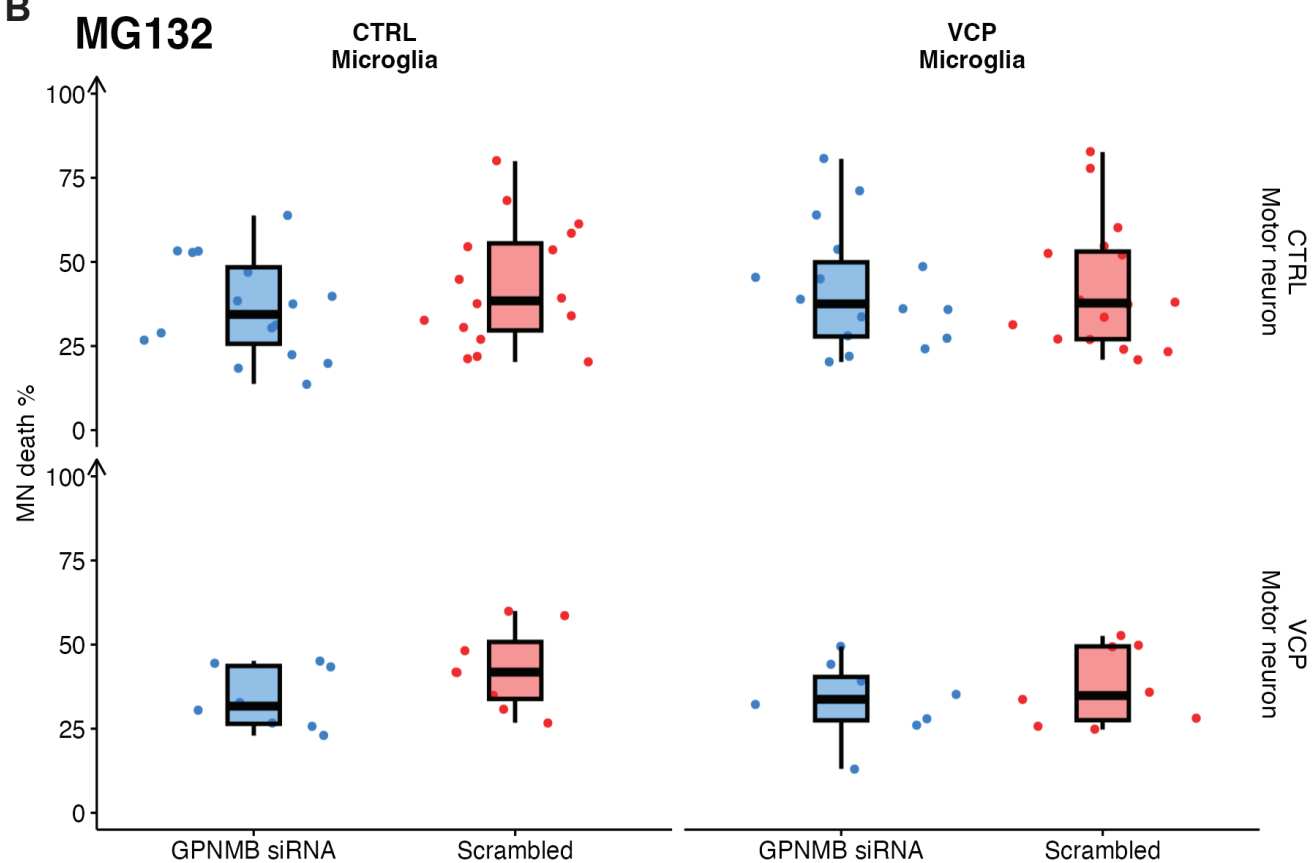

Supplement: Supplementary file 8 — Supplementary Material 8: Figure S8: Microglial GPNMB knockdown has no effect on motor neuron survival. Quantification of control and VCP mutant motor neuron survival under basal conditions (A) or after treatment with MG132 (B) for 21 hours after pretreatment with conditioned media from healthy control or VCP mutant microglia treated with scrambled control or GPNMB targeting siRNA. Scale bar: 200μm. Data are from 4 lines from 2-4 differentiations. Stats from generalised linear model accounting for cell line and experimental repeat. [file 13024_2024_773_MOESM8_ESM.pdf]

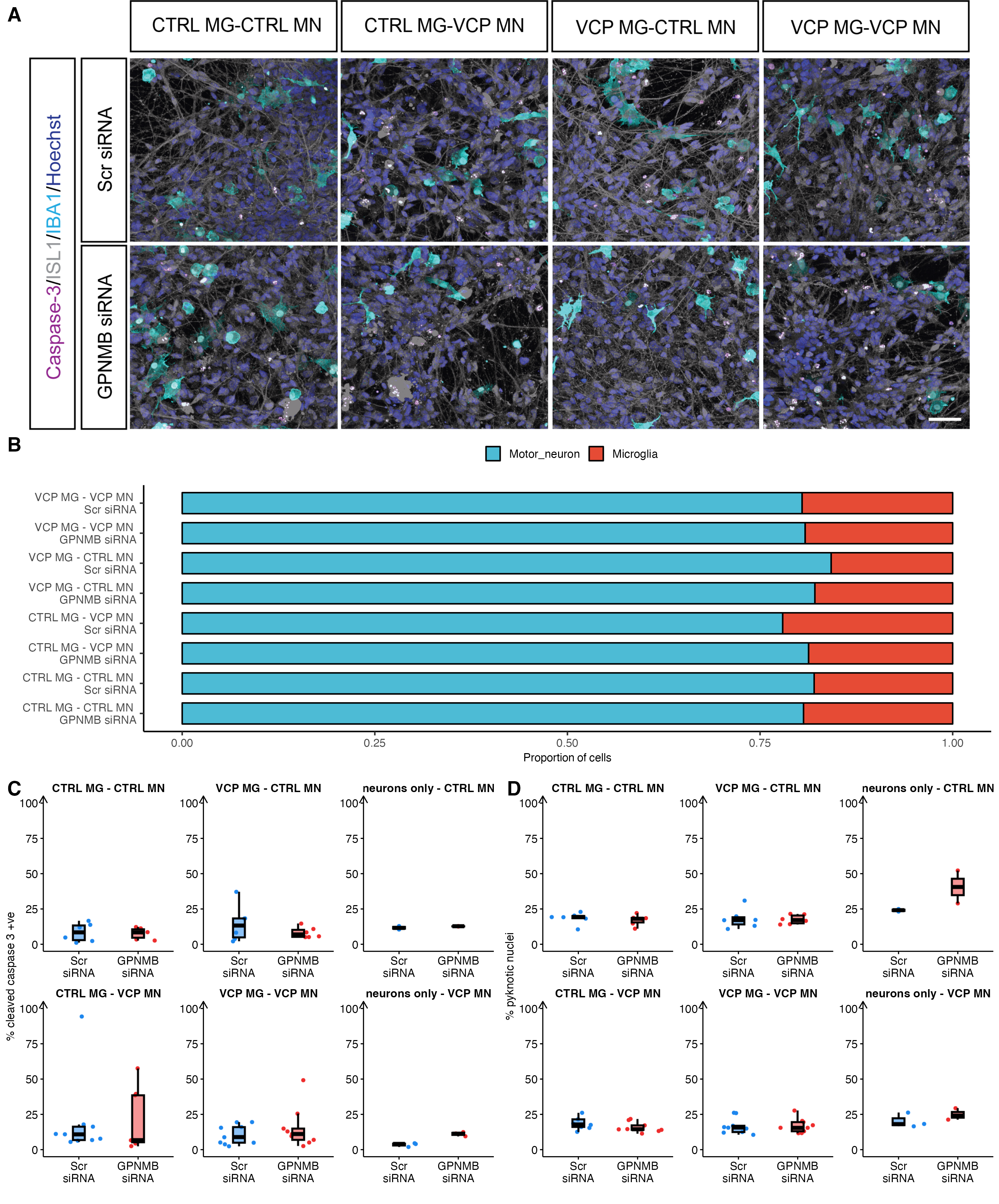

Supplement: Supplementary file 9 — Supplementary Material 9: Figure S9. Microglial GPNMB knockdown has no effect on motor neuron survival. (A) Representative images of immunofluorescence for cleaved caspase-3 (magenta), ISL1 (grey), IBA1 (cyan) and staining for DAPI (blue) from control and VCP mutant microglia and motor neuron co-cultures treated with scrambled or GPNMB siRNA. Scale bar: 50 um. (B) Quantification of proportions of ISL1 positive motor neurons and IBA1 positive microglia in control and VCP mutant microglia and motor neuron co-cultures treated with scrambled or GPNMB siRNA. Quantification of cleaved caspase-3 positive (C) and pyknotic motor neurons in control and VCP mutant microglia and motor neuron co-cultures treated with scrambled or GPNMB siRNA. Data are N =3 motor neuron and N =3 microglia lines per genotype from 1 differentiation. [file 13024_2024_773_MOESM9_ESM.png]
